# Enhanced Thermoelectric Performance of PVA-Based Ionogels: Tailoring Crystallinity via Additives for Advanced Waste Heat Recovery

**DOI:** 10.1021/acsami.5c08724

**Published:** 2025-06-21

**Authors:** Ling-Chieh Lee, Shao-Huan Hong, Min-Su Kim, U-Ser Jeng, Chia-Hsin Wang, Shih-Huang Tung, Keun Hyung Lee, Cheng-Liang Liu

**Affiliations:** † Department of Materials Science and Engineering, 33561 National Taiwan University, Taipei 10617, Taiwan; ‡ Department of Chemistry and Chemical Engineering, 26718 Inha University, Incheon 22212, Republic of Korea; § 57815 National Synchrotron Radiation Research Center, Hsinchu 30076, Taiwan; ∥ Institute of Polymer Science and Engineering, 33561 National Taiwan University, Taipei 10617, Taiwan; ⊥ Advanced Research Center for Green Materials Science and Technology, National Taiwan University, Taipei 10617, Taiwan

**Keywords:** ionic thermoelectrics, thermopower, ionic liquid, ionogel, low-grade heat harvesting

## Abstract

Converting low-grade waste heat into electricity is crucial for green energy. This study introduces an innovative approach using poly­(vinyl alcohol) (PVA)-based ionogels incorporating 1-ethyl-3-methylimidazolium dicyanamide ([EMIM]­[DCA]) and specific additives: 2-carboxyphenylacetic acid (H), 2-sulfobenzoic acid (S), and 2-carboxyphenyl phosphate (P)). These additives enable successful tailoring of the crystallinity, leading to a substantial increase in the ionic figure-of-merit (z*T*_i_), from 0.006 for the PVA ionogel to 0.27 for the ionogel with P additives. Furthermore, the P-additive ionogels exhibit excellent mechanical properties, with a tensile stress of 1.75 MPa and a strain of 460%. A four-pair ionic thermoelectric capacitor made from these ionogels generates 0.33 V and achieves a power output of 2.4 mW m^–2^. This advancement significantly improves the thermoelectric performance of PVA ionogels, aiding in efficient waste heat utilization and sustainable energy development.

## Graphical abstract

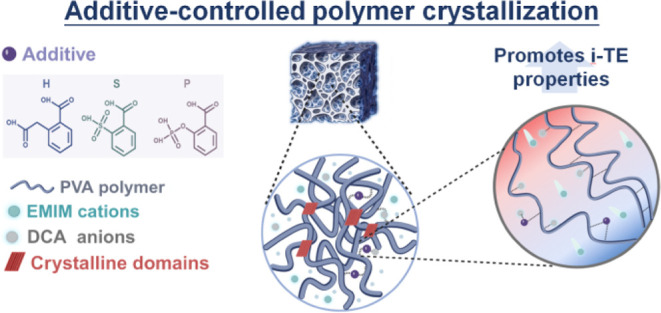

## Introduction

Due to the global energy shortage, developing green energy solutions is increasingly critical. Thermoelectric (TE) recycling, which converts low-grade waste heat into electrical energy, shows particular promise.^1–3^ TE materials are broadly classified into two types: electronic and ionic.^4–8^ While conventional TE generators use electrons or holes as charge carriers for continuous power generation, the recently emerging ionic thermoelectric capacitors (ITECs) operate based on ion diffusion in ionic thermoelectric (i-TE) materials,^9^ which offer a significant advantage in output voltage (millivolts to tens of mV K^–1^)^10–12^ compared to conventional electronic TE materials (tens to hundreds of μV K^–1^).^13,14^ This higher output voltage is especially advantageous for self-powering wearable devices. The performance of an i-TE material fundamentally relies on the Soret effect (or thermophoresis), which involves the spontaneous movement of ions in response to a thermal gradient.^15,16^ The efficiency of an i-TE material is quantified by its ionic thermopower (*S*_i_) and defined as eq 1:

$$S_{i}=-\frac{\Delta V}{\Delta T}=-\frac{V\left(T_{H}\right)-V\left(T_{C}\right)}{T_{H}-T_{C}}=\frac{\left({Ŝ}_{+}D_{+}\right)-\left({Ŝ}_{-}D_{-}\right)}{e\left(D_{+}+D_{-}\right)}$$ 1

Here, *V*(*T*_H_) represents the voltage of the hot electrode at temperature *T*_H_, *V*(*T*_C_) denotes the voltage of the cold electrode at temperature *T*_C_, *e* is the elementary charge, Ŝ signifies the Eastman entropy of transfer, *D* is the mass diffusion coefficient, and the subscripts + and – represent the cations and anions, respectively.^17,18^ It is important to note that, in accordance with the formula for the *S*_*i*_, a negative value denotes n-type behavior, while a positive value signifies p-type behavior.^16^ The performance of i-TE materials is characterized by the dimensionless ionic figure-of-merit (*zT*_*i*_) and defined as eq 2:

2 $${\mathrm{zT}}_{i}=\frac{S_{i}^{2}\sigma_{i}T}{\kappa }$$

Here, *S*_i_ is ionic thermopower, σ_i_ denotes the ionic conductivity, κ is the thermal conductivity, and *T* is absolute temperature, respectively. To achieve a high *zT*_*i*_, it is crucial to simultaneously enhance both *S*_i_ and σ_i_ while minimizing κ.^19^ Compared to their liquid counterparts, several quasi-solid-state i-TE materials have been effectively designed and developed. These materials offer enhanced flexibility and higher ionic thermopower due to more complex interactions among ions, polymers, and the surrounding media.^20^ Quasi-solid-state^21,22^ materials are commonly used in batteries and supercapacitors because of their unique mix of solid- and liquid-like properties.^23^ These materials typically consist of an electrolyte with a polymer matrix that provides structural integrity and mechanical stability. The conductivity of the electrolyte is enhanced by incorporating inorganic salts, polyelectrolytes, or ionic liquids (ILs). Among these, ionogels stand out due to their nonvolatility and excellent thermal stability. In recent years, strategies have been developed to fabricate IL-based ionogels, including the use of ILs with poly­(vinylidene fluoride-*co*-hexafluoropropylene) (PVDF-HFP)^24^ and other polymer matrices like cellulose combined with SiO_2_ nanoparticles^25^ and polyurethane (PU)^26^ – see Table S1. While these materials exhibit excellent i-TE properties, their mechanical properties often fall short of the required elasticity and stretchability for wearable devices.

Poly­(vinyl alcohol) (PVA), a hydrophilic polymer with multiple hydroxyl groups, exhibits inherent crystallization behavior, which can play a crucial role in enhancing its mechanical properties. For instance, Huang et al. fabricated PVA hydrogels containing 40% 1-ethyl-3-methylimidazolium dicyanamide ([EMIM]­[DCA]) and measured a *S*_i_ of approximately 4 mV K^–1^ and a tensile strength of 2.03 MPa.^27^ However, to the best of our knowledge, there are no other reports on PVA-based ionogels in the i-TE field expect for the above work by Huang et al. This scarcity may be attributed to the reduced i-TE properties associated with the semicrystalline structure of PVA and the presence of hydroxyl groups in its side chain,^28^ which tend to crystallize when immersed in salts, potentially impeding ion transport.^16^ To address this problem, a novel approach is introduced that involves incorporating additives with various functional groups to regulate crystallinity. The addition of an IL is crucial to impart conductivity. By incorporating additives, a novel pathway for the PVA-ionogel system has been established.

This study focuses on developing ionogels using PVA, additives, and [EMIM]­[DCA] as the conductor. The additives improve chemical compatibility and molecular interaction with the PVA polymer. Additionally, the electronegative oxygen atoms within the additives facilitate interactions with negatively charged molecules, such as DCA cations. Furthermore, the organic solvents were selected for their high solvating power. They also show excellent thermal and environmental stability, as well as enhance mechanical stability through physical interaction with polymer networks. The resulting PVA ionogels with the additives maintain a high stress (1.75 MPa) and 460% strain. In terms of i-TE properties, ionogels with additives achieved the highest key parameters (*S*_i_ = 9.5 mV K^–1^, σ_i_ = 18.5 mS cm^–1^, κ = 0.19 W m^–1^ K^–1^). This enhancement can be attributed to the additives, which effectively control PVA crystallinity and interact with DCA anions through their electron-withdrawing groups. ITEC device achieved a maximum power output of 6.8 mW m^–2^, and a 4-pair ITEC module produced a voltage output of 0.33 V. These results demonstrate that using specific additives to control crystallinity and enhance i-TE properties represents a significant advancement in i-TE performance, paving the way for future research and development in advanced i-TE materials and wearable devices.

## Results and Discussion

### Structure and Composition of i-TE Ionogels

The ionogels are composed of PVA polymer as the matrix, ionic liquid (IL) [EMIM]­[DCA] as the ionic conductor, and three different additives serving as crystallinity controllers. The chemical structures of these components are shown in Figure 1a. The additives used include 2-carboxyphenylacetic acid (H), 2-sulfobenzoic acid (S), and 2-carboxyphenyl phosphate (P), respectively. To systematically categorize the gels and elucidate the variations shown in Figure 1b, the following nomenclatures are used: Organogels without additives are referred to PVA organogel. In contrast, organogels containing additives are denoted as PVA:X, where X represents the specific additives: 2-carboxyphenylacetic acid (PVA:H), 2-sulfobenzoic acid hydrate (PVA:S), and 2-carboxyphenyl phosphate (PVA:P). Additionally, the immersion of each organogel in [EMIM]­[DCA] IL converts it into an ionogel, represented as PVA:X-IL. Figure 1c illustrates the preparation of PVA organogels using PVA and additives through physical crosslinking, as detailed in the methods section. This depiction indicates that the PVA chains are solvated by the solvent in the presence of the additives. Additives (H, S, and P), which possess electron-withdrawing groups, enhance the dispersion of PVA in the solvent, thereby reducing crystalline points. Upon immersing in the IL, the ions interact strongly with the solvent, causing the dimethyl sulfoxide (DMSO) or N,N-Dimethylformamide (DMF) molecules to be expelled from between the polymer chains, leading to the reduction in the solubility of PVA chains, analogous to the salting-out effect observed in aqueous solutions. Consequently, hydrogen bonds form between hydroxyl groups on PVA, resulting in the aggregation or crystallization of the polymer chains. Figure 1d illustrates the interplays among the PVA chains, ions, and additives, and their collective influence on the ionic thermoelectric (i-TE) properties of the ionogels within crystalline regions. The additives disrupt the regular packing of PVA chains, resulting in reduced overall crystallinity and enhanced cation transport. The DCA anions interact with hydroxyl groups on the PVA chains and additives, allowing EMIM cations to move more freely under a temperature gradient. This enhances the concentration gradient between cations and anions, thus improving the i-TE properties of the ionogels.

**Figure 1:**
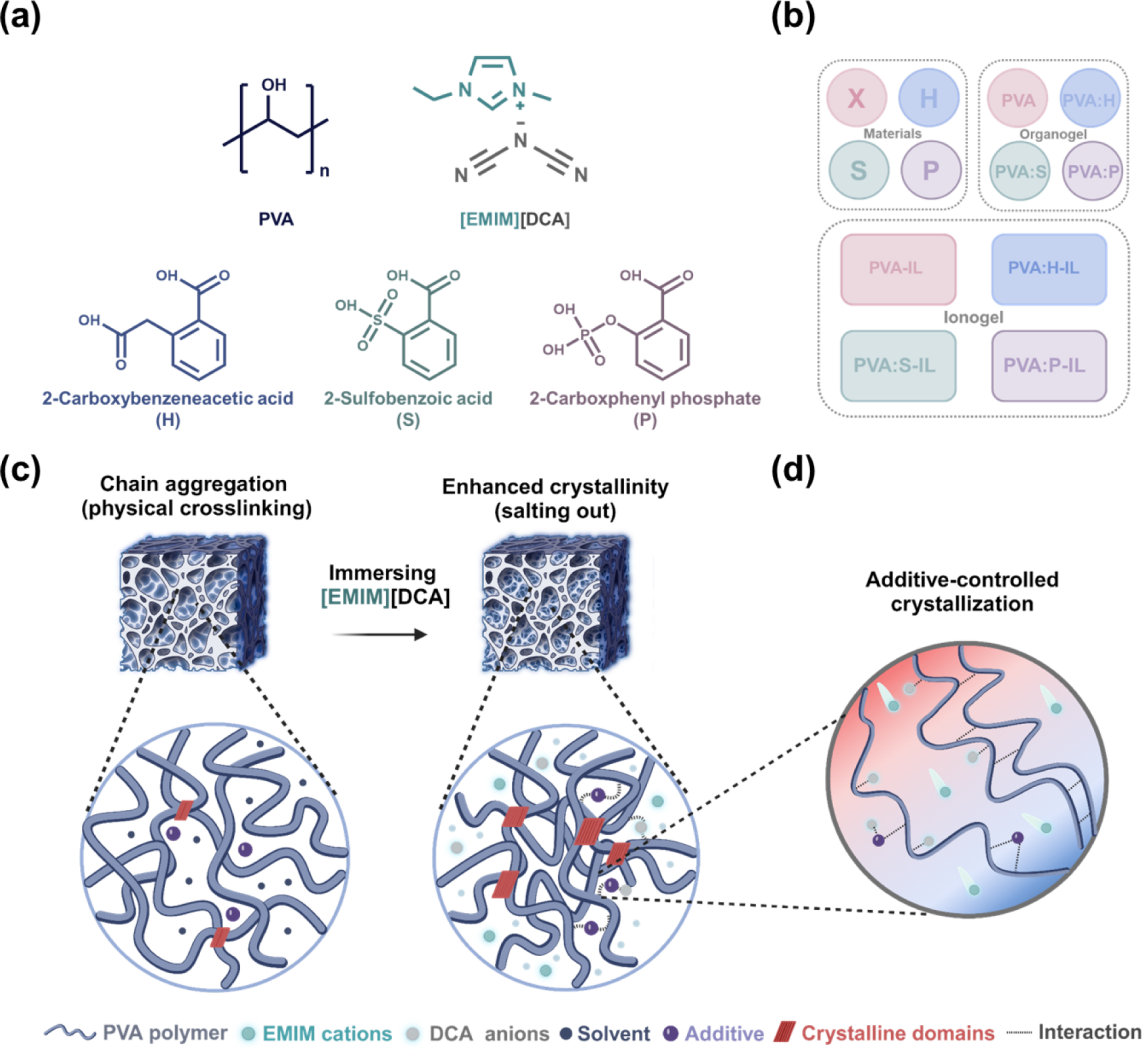
Schematic representation of the investigated i-TE materials and ionic interaction in PVA-based ionogels. (a) Chemical structures of the gel matrix (PVA), ionic conductor ([EMIM]­[DCA]), and additives (H, S, and P). (b) Defined gel names and categorization into three types: materials, organogels, and ionogels. (c) Schematic of the ionic interaction within the PVA gels before and after immersion in the IL. (d) Schematic illustrating the cation transport to amorphous regions under a temperature gradient.

### Morphological Characterization of PVA-Based Gels

The surface, cross-sectional, and microstructural morphologies of organogels and ionogels, as revealed by the scanning electron microscope (SEM) images, are exhibited in Figures 2 and S1. In Figure 2a**–**d, all organogels demonstrate robust structural integrity without wrinkles. Notably, the SEM images in Figure 2b**–**d indicate that introducing additives disrupts the compact structure of the PVA chains, increasing the free volume resulting in distinct black and white regions. The PVA:H and PVA:S exhibit slightly uneven textures, while PVA:P organogel shows the most pronounced effect with irregular textures. The cross-sectional morphologies of the organogels, shown in the Figure S1a, indicate that the PVA organogel has a denser structure compared to the others. In contrast, shown in the Figures S1b, S1c and S1d, the presence of additives in PVA:H, PVA:S, and PVA:P result in a less-dense morphology, respectively. The surface of the ionogels, shown in Figure 2e**–**h, are rougher compared to the organogels. In Figure 2e, PVA-IL displays a highly wrinkled structure, indicating a local aggregation of PVA chains. Conversely, PVA:H-IL and PVA:S-IL exhibit fewer wrinkles, indicating that the local aggregation was alleviated by the H or S additive (Figure 2f,g). In Figure 2h, the surface of PVA:P-IL was only slightly wrinkled, implying that the P additive has a stronger effect on preventing PVA chains from aggregation. The observations are corroborated by the energy-dispersive X-ray spectroscopy (EDS) results shown in Figures S2 and S3, which provide a detailed characterization of the PVA:P-IL by elemental mapping. The EDS maps in Figure S2b–g depict the distribution of carbon, oxygen, nitrogen, sulfur, and phosphorus elements, showing a homogeneous distribution of nitrogen elements (highlighted in orange in Figure S2e), which indicates effective dispersion of the IL ([EMIM]­[DCA]) and DMF solvent within the gel. At higher magnification in Figure S3, PVA:P-IL reveals two distinct distributions: the white region represents polymer-rich areas, while black region represents polymer-poor areas, as shown in Figure S3a. Notably, the phosphorus elements (highlighted in green in Figure S3c) are distributed in the polymer-rich regions, suggesting a significant interaction between the PVA and P additive, which provides a better stabilization for PVA chains in the solvent. These observations indicate the substantial influence of additives on the microstructural integrity of PVA polymer.

**Figure 2:**
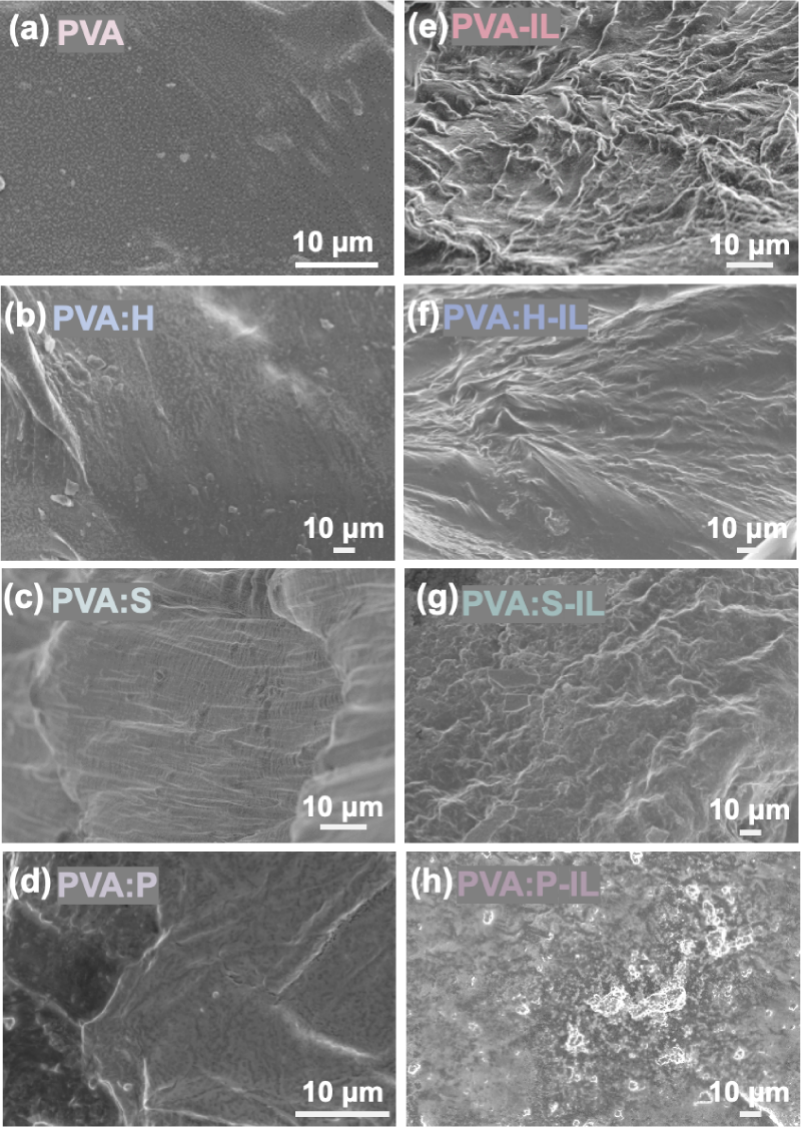
Morphological characterization of the PVA-based gels. SEM images of various PVA-based gels: (a) PVA organogel, (b) PVA:H, (c) PVA:S, (d) PVA:P, (e) PVA-IL, (f) PVA:H-IL, (g) PVA:S-IL, and (h) PVA:P-IL. Scale bar: 10 μm.

### Characterizing Interactions in PVA-Based Gels

To analyze the potential binding sites of additives with DCA anions and illustrate the charge distribution, an electrostatic potential (ESP) map was generated – see Figure 3a. It reveals that the regions surrounding the oxygen atoms in the carboxylic acid of the H additive, the sulfur atoms in the sulfonic acid of the S additive, and the phosphorus atoms in the phosphate of the P additive exhibit the most negative electrostatic potential. In contrast, for DCA anions, the most positive electrostatic potential is located at the center. Therefore, the regions with the most negative electrostatic potential are likely to interact with the oxygen, sulfur, and phosphorus atoms of the respective additives. To clearly elucidate the interactions between DCA and additives, the binding energies of the DCA anions with additives were subsequently calculated using density functional theory (DFT) – see Figure 3a. The results indicate that the interaction affinity of H:DCA is – 24.12 kJ mol^–1^ and S:DCA is – 27.98 kJ mol^–1^, respectively, both of which are weaker compared to P:DCA which has an affinity of – 40.52 kJ mol^–1^. This suggests that the interaction between DCA anions and the P additive is the strongest among all the additives.

**Figure 3:**
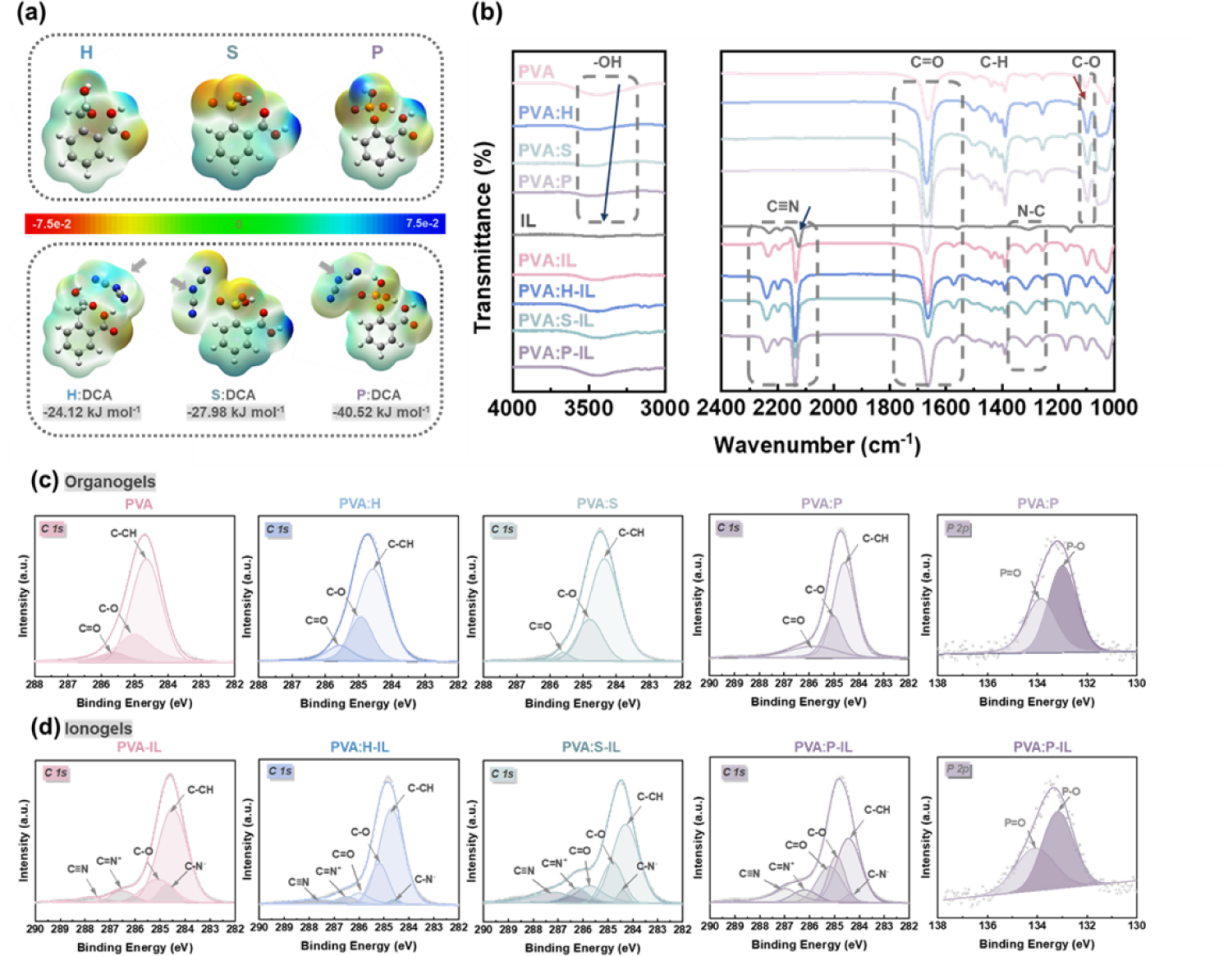
Characterization of the interactions in PVA-Based gels using ESP, FT-IR, and XPS. (a) Electrostatic potential (ESP) map for different additives with specific additives: H, S, P, and their corresponding binding energies with DCA anions (H-DCA: – 24.12 kJ mol^–1^, S-DCA: – 27.98 kJ mol^–1^, P-DCA: – 40.52 kJ mol^–1^). (b) FT-IR spectra of the PVA organogels, ionogels, and ILs. The left plot shows the transmittance for the wavenumber range 4000–3000 cm^–1^. The right plot shows the transmittance for the wavenumber range 2400–1000 cm^–1^. (c) XPS spectra of the C 1s and P 2p regions for PVA-based organogels (top row) and (d) PVA-based ionogels (bottom row).

To verify the binding sites of additives with DCA anions and to investigate other interactions involving PVA chains, additives, and ILs, Fourier transform-infrared (FT-IR) and X-ray photoelectron spectroscopy (XPS) spectra were employed. These techniques are sensitive to vibrations of unique functional groups – see Figure 3b,c. Figure 3b shows the FT-IR spectra of PVA, PVA:H, PVA:S, and PVA:P organogels (above the IL spectrum). The peak observed at 3439 cm^–1^ corresponds to the **–**OH stretching vibration of the PVA polymer, while the peaks at 1664, 1437, and 1102 cm**^–^** ^1^ are due to C = O stretching vibrations of DMF, the C**–**H stretching vibrations of the PVA polymer, and the C**–**C**–**O stretching vibrations promoting interaction between PVA chains in the amorphous region.^29^ Note that the – OH peak in the PVA organogel shows a blue shift from 3439 to 3495 cm^–1^ upon introducing the P additive. This shift is attributed to interactions between the hydroxyl groups in the PVA polymer and the additives. The polar and hydrogen bonding interactions of the oxygen-containing groups in the additives drive these interactions. In addition, this interaction results in an averaging of the electron cloud density between the hydroxyl groups in the PVA polymer and the oxygen groups in the additives, where the most significant effect is observed for the P additive.^30^ The increased intensity of the C = O peak at 1664 cm^–1^ indicates a higher concentration of carboxylic acid groups from the additives, which suggests effective dispersion of the additive within the PVA matrix. Furthermore, the intensity of the amorphous peak at 1102 cm^–1^ increases and displays a red shift due to the introduction of the additive. This higher intensity and shift are attributed to the interaction between the PVA polymer and the additives, which disrupts the aggregation of the PVA polymer and promotes the formation of an amorphous region.^29^ Moreover, this interaction significantly increases the density of the amorphous region within the polymer network, which was observed using SEM (Figure 2d), where irregular textures were evident in PVA:P. Figure 3b displays the FT-IR spectra of pure IL and all ionogels (below the IL spectrum). In the pure ILs, the peak at 2124 cm^–1^ corresponds to the C≡N stretching vibration of DCA anions, while the peak at 1306 cm**^–^** ^1^ is associated with the N**–**C stretching vibrations of EMIM cations.^27^ The C≡N stretching peak (associated with DCA anions) shifts from 2124 cm^–1^ in pure IL to 2134 cm^–1^ in PVA-IL. This blue shift indicates binding of DCA anions to the hydroxyl groups in the PVA polymer. Additionally, for the PVA:H-IL, PVA:S-IL, and PVA:P-IL ionogels, this peak remains around 2134 cm^–1^. Furthermore, all ionogels exhibit a blue shift in the N**–**C bond (due to the EMIM cation) compared to the pure IL. This suggests that electron transfer may occur upon the introduction of EMIM cations into the system. This electron transfer process is facilitated by the chaotropic properties of the EMIM cation, which potentially enables these cations to be transported in the amorphous regions.^31^

Further evidence is provided by the XPS results shown in Figure 3c. In the C 1s spectrum of all studied PVA organogels, three peaks (284.6 eV, 285.0 eV, 285.6 eV) are observed, which correspond to the C**–**O, C = O, and C**–**C/C**–**H groups, respectively.^32,33^ The introduction of additives (PVA:H, S, or P) increases the peak intensity associated with C = O, which is attributed to the carboxylic acid functional group in the additives. In addition, upon immersing in the IL, all ionogels exhibit additional peaks which correspond to C**–**C, C**–**N^–^, C**–**O, C = O, C = N^+^, and C≡N. Notably, the C = N^+^ (associated with EMIM cations) peak shifts to lower binding energies from 286.5 to 286.5, 286.3, and 286.2 eV for PVA-IL, PVA:H-IL, PVA:S-IL, and PVA:P-IL, respectively. This shift is attributed to increased electron density in the EMIM cations within the PVA:P-IL system. This phenomenon occurs because the P additive exhibits stronger interactions with PVA chains, which hinders the crystallization of PVA and leads to a higher density in the amorphous regions. Furthermore, the increased amorphous content facilitates easier electron exchange between EMIM cations and exposes hydroxyl groups in the PVA chain. The shifts observed in the C≡N peak to lower binding energies, from 287.8 to 287.6, 287.2, and 287.0 eV for PVA-IL, PVA-IL, PVA:H-IL, PVA:S-IL, and PVA:P-IL respectively, are due to the interaction between the C≡N of the DCA anions and the functional groups of the additives. Moreover, additives with different electronegative oxygen groups facilitate varying interactions with the negatively charged DCA anions, which leads to different shifts. These findings are consistent with the results from the DFT analyses. In addition, the P 2p spectrum supports this conclusion, with the PVA:P and PVA:P-IL deconvoluted into two peaks: 133.0 and 133.2 eV for PVA:P, and 133.9 and 134.1 eV for PVA:P-IL, corresponding to P**–**O and P = O bonds, respectively. The shift to higher binding energies in P–O and *p* = O suggests interaction with DCA anions that leads to electron loss.

Combining the DFT, FT-IR, and XPS analyses reveals that different additives affect the hydrogen bonding properties within the PVA polymer. This effect is particularly pronounced for the P additive due to its strong electron-withdrawing groups, which potentially expand the amorphous regions within the gels. DCA anions are expected to interact with the hydroxyl groups of the PVA chains, and DFT and XPS analysis shows that different additives impact the degree of this interaction. The EMIM cations may exhibit small interactions within the system due to their chaotropic effect, which disrupts the hydrogen bonds in the amorphous regions and facilitates their transport within the system. Until now, validation of these interactions between the ILs, additives, and matrix was done solely through FTIR and XPS. Next, X-ray diffraction (XRD) will be utilized to thoroughly examine crystallinity.

### Characterization of the Microstructure in PVA-Based Gels

The XRD profiles of the PVA-based organogels and ionogels are shown in Figure 4. The diffraction pattern of PVA organogel reveals two peaks at 2θ = 19.3° and 22.7°, assigned to the (101) and (200) planes, respectively.^34^ Introducing the additives into the PVA organogel results in similar intensity, but the peaks at 19.3° and 22.7° become broader, which indicates increased peak width. This broadening is likely due to the additives disrupting some of the hydrogen bonds between polymer chains, which increases free volume and enhances the amorphous regions. In comparison, the ionogels exhibit new peaks at 2θ = 16.2° and 40.3° that correspond to the (100) and (111) planes, respectively,^35^ while the organogels do not. Furthermore, all ionogels show sharper peaks and a higher intensity than the organogels, which suggests an improvement in crystallinity upon immersion in the IL. This effect occurs because the ions promote the aggregation of PVA chains, a process facilitated by the reduced solubility of PVA in the solvent due to the IL. As a result, hydrogen bonds form between hydroxyl groups on PVA, which facilitates the crystallization of the polymer chains. These results are consistent with the SEM findings. In contrast, the ionogels with additives, especially those containing P, show broader peaks than those without additives. In other words, the additives can suppress the PVA crystallization even in the presence of the IL. The degree of crystallinity (*X*_c_) of the organogels was approximated using the XRD profiles in Figure 4 based on the ratio between the area of the crystalline reflection in the 2θ ranging from 18.0 to 22.7° and the area of the total diffraction profile of the gels. This can be formulated as follows eq 3:^36^

3 $${Χ}_{c}=\frac{A_{C}}{A_{T}}\times \mathrm{100\%}$$

**Figure 4:**
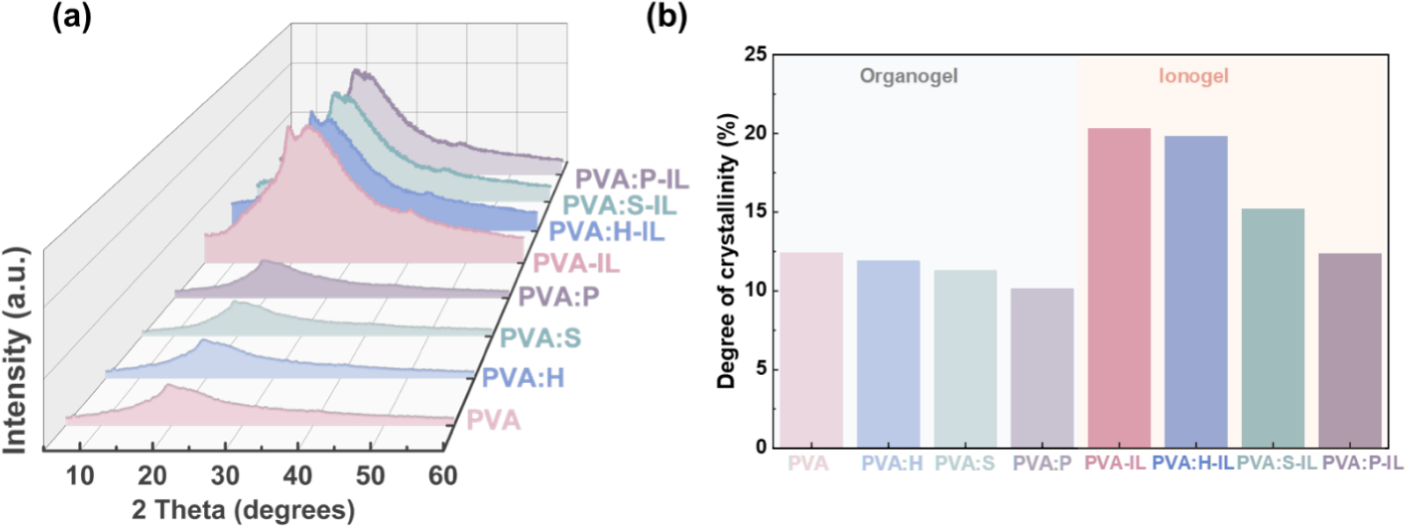
X-ray diffraction (XRD) analysis and crystallinity comparison of the PVA-based gels. (a) XRD patterns of various PVA-based organogels and ionogels. (b) Comparative column graph showing the degree of crystallinity for organogels and ionogels.

Here, *A*_C_ and *A*_T_ denote the areas of crystalline peaks and the total area of amorphous and crystalline peaks, respectively, and the fitting XRD deconvolutions for organogels and ionogels are shown in Figure S4. Figure 4b shows that *X*_c_ decreases from 12.4% in PVA organogels to 10.2% in PVA:P when additives are introduced. The crystallinity increases upon immersion in the IL, reaching a maximum of 20.3% in PVA-IL and decreasing to 12.4% in PVA:P-IL – see Table S2. This finding aligns well with the morphology observed using SEM (see Figure 2a,e), where the PVA organogel displays a smoother surface, which suggests it is less crystallized than PVA-IL. Compared to PVA-IL, the PVA:X-IL samples show fewer wrinkles, which suggests decreased crystallinity. Furthermore, the lower *X*_c_ in PVA:X-IL implies enlarged amorphous regions, especially with the addition of the P additive, corroborating the analysis above.

In order to investigate the impact of different additives on the nanostructure of the organogels and their behavior when immersed in IL, a structural analysis of the gels was conducted using small-angle X-ray scattering (SAXS). The SAXS data were fit using the Beaucage equation, which accommodates multiple interdependent structural levels (equation S2, see detail in Supporting Information files). According the Beaucage equation, *G*_*i*_ represents Guinier’s pre-exponential factor, while *B*_*i*_ corresponds to the power law pre-exponential factor. *R*_*gi*_ stands for the radius of gyration, and *P*_*i*_ denotes the fractal dimension.^37^ In this context, *i* is associated with different levels of structural organization: *i* = 1 corresponds to the global structure, and *i* = 2 signifies the characterization of local structures. Figures 5 and S5 display the measured scattering curve and the corresponding fitting results, while Table S3 summarizes the fitting results for the gels. For all gels, as shown in Figures 5 and S5, the unified fitting approach reveals the presence of two discernible power-law regions that correspond to two distinct structural levels – each manifesting within specific *q*-ranges: large-scaled agglomerates within the *q*-range of 0.005–0.03 Å^–1^ (1st level) and small-scaled aggregates in the *q*-range of 0.03–0.1 Å^–1^ (2nd level). At the first level, a power law analysis yields an exponent of *P*_1_ = 2, which suggests a mass fractal structure, i.e., a denser agglomeration of the polymer.^38^ At the second level the power law analysis yields an exponent of *P*_2_ = 4 due to the primary particles aggregated by PVA chains, which are the building blocks of the agglomerates.^38,39^ Figure 5e illustrates the hierarchical structure of polymer agglomerates and highlights two levels of structures and their respective radii of gyration (*R*_g1_ and *R*_g2_). The agglomerate structure, depicted as a mass-fractal structure with *R*_g1_, corresponds to the larger clusters formed by the primary particles with *R*_g2_. Although the power law exponents for the two structural levels are consistent, the *R*_g_s of the gels varies with additives and the IL immersion. For the PVA organogel, the data fit yields *R*_g1_ = 44.6 nm for the agglomerates and *R*_g2_ = 4.5 nm for the primary particles, respectively. Both *R*_g1_ and *R*_g2_ decrease with the introduction of additives. As shown in Figure 5b and Table S3, *R*_g1_s of PVA:H, PVA:S, and PVA:P organogels drop to 32.1, 34.8, and 33.7 nm, while the *R*_g2_s values drop to 4.0, 3.8, and 3.8 nm, respectively. This is attributed to the effects of the additives that facilitate the dispersion of the PVA chains in the solvent, which reduces the size of the agglomerates and primary particles.^40^ As expected, compared to the PVA organogel, *R*_g1_ and *R*_g2_ of the PVA-IL ionogel increase to 123.1 and 4.9 nm, respectively, due to the IL-induced aggregation and crystallization of PVA chains. As shown in Figure 5d, a similar effect of the additives appears in ionogels, where the *R*_g1_s values of PVA:H-IL, PVA:S-IL, and PVA:P-IL decrease to 58.1, 46.8, and 45.4 nm while *R*_g2_s drop to 4.9, 4.5, and 4.1 nm, respectively. The results are in line with the XRD analysis.

**Figure 5:**
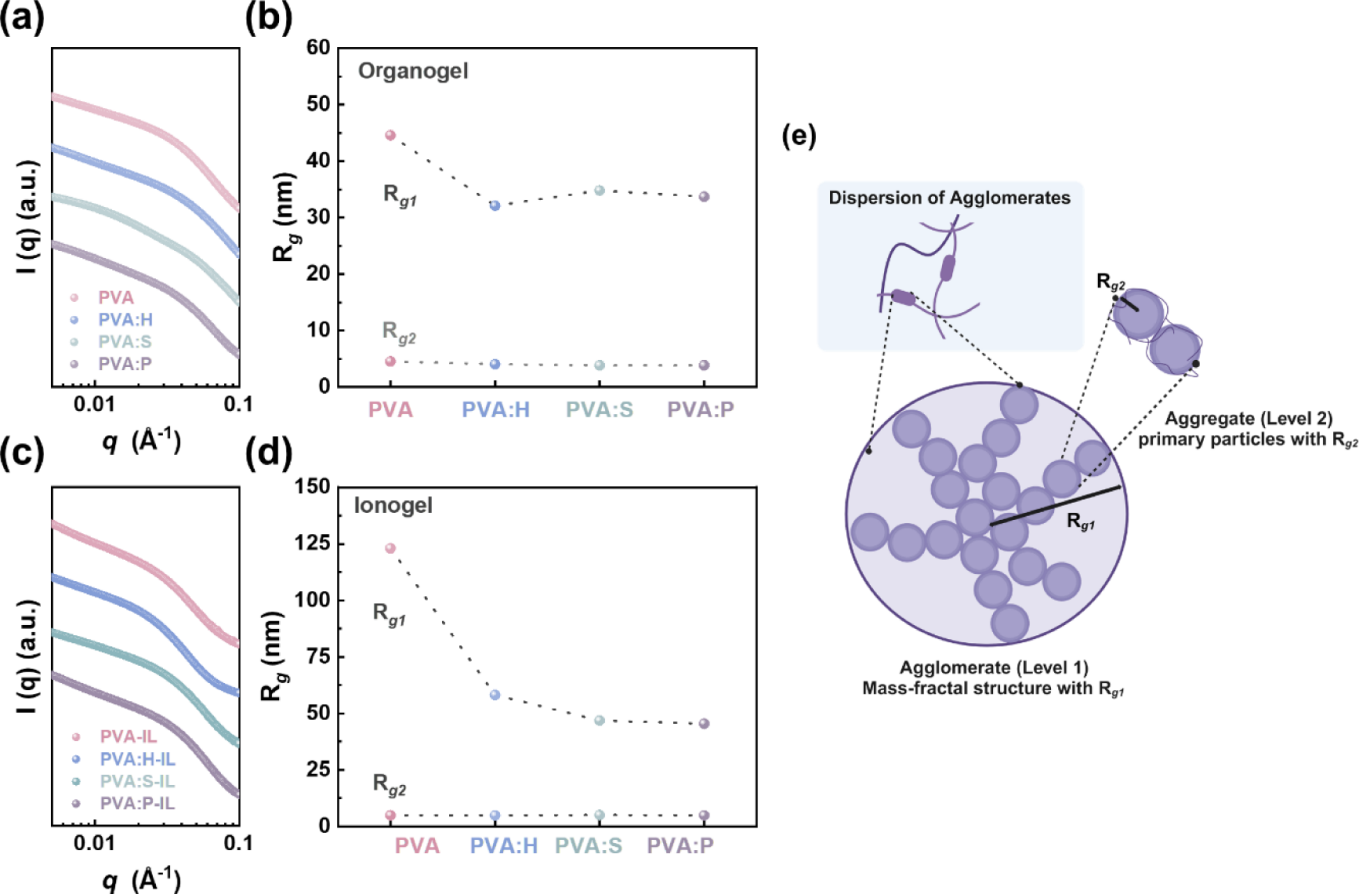
Characterization of the microstructure in PVA-based gels. SAXS patterns of the PVA-based (a) organogels and (c) ionogels. Radius of gyration of PVA-based (b) organogels and (d) ionogles. (e) Schematic depicting the hierarchical structure of polymer agglomerates, highlighting two levels of structural organization and their respective radii of gyration (*R*_*g1*_ and *R*_*g2*_).

### Mechanical and Rheological Properties in PVA-Based Gels

The mechanical properties of the organogels and ionogels are crucial for their use in stretchable device applications. In order to gain further insights into the thermorheological properties of the organogels and ionogels, the dynamic storage modulus (*G’*) and loss modulus (*G”*) were thoroughly examined across a temperature spectrum ranging from 20 to 50 °C – see Figure 6a,b. Notably, for both the organogels and ionogels, the *G’* values consistently exceed the *G”* values across the entire temperature range. This indicates that all gels exhibit quasi-solid behavior rather than a liquid-like state,^41^ which demonstrates their stability with respect to temperature fluctuations. Moreover, the *G’* of the ionogels exceeds that of the organogels because immersion in ILs improves crystallinity. In contrast, introducing the additive enlarges the amorphous regions of the ionogels and decreases *G’*. Moreover, the mechanical properties of organogels and ionogels, upon introducing different additives, are assessed through stress–strain curves – see Figure 6c,d. In Figure 6c, the PVA organogel exhibits a high tensile strength of 1.5 MPa and a break elongation of 280%. The organogels with the additives maintain a similar tensile strength, but the additives enhance their elongations in the order: PVA < PVA:H < PVA:S < PVA:P. This confirms that the introduction of additives modifies the hydrogen bond in the PVA polymer and expands the amorphous regions, which in turn improves the elongation properties. Furthermore, the ionogels exhibit higher tensile strength due to molecular interactions and increased crystallinity–among which PVA-IL and PVA:H-IL exhibit the highest stress. Additionally, the ionogels with additives show increased elongation due to the enlargement of the amorphous regions, which enhances flexibility and reduces brittleness. In Figure 6d, PVA:P-IL exhibits a high tensile strength of 1.75 MPa, accompanied by a break elongation of 460%. These results are consistent with the XRD findings, which show that PVA:P-IL features more amorphous regions than PVA-IL.

**Figure 6:**
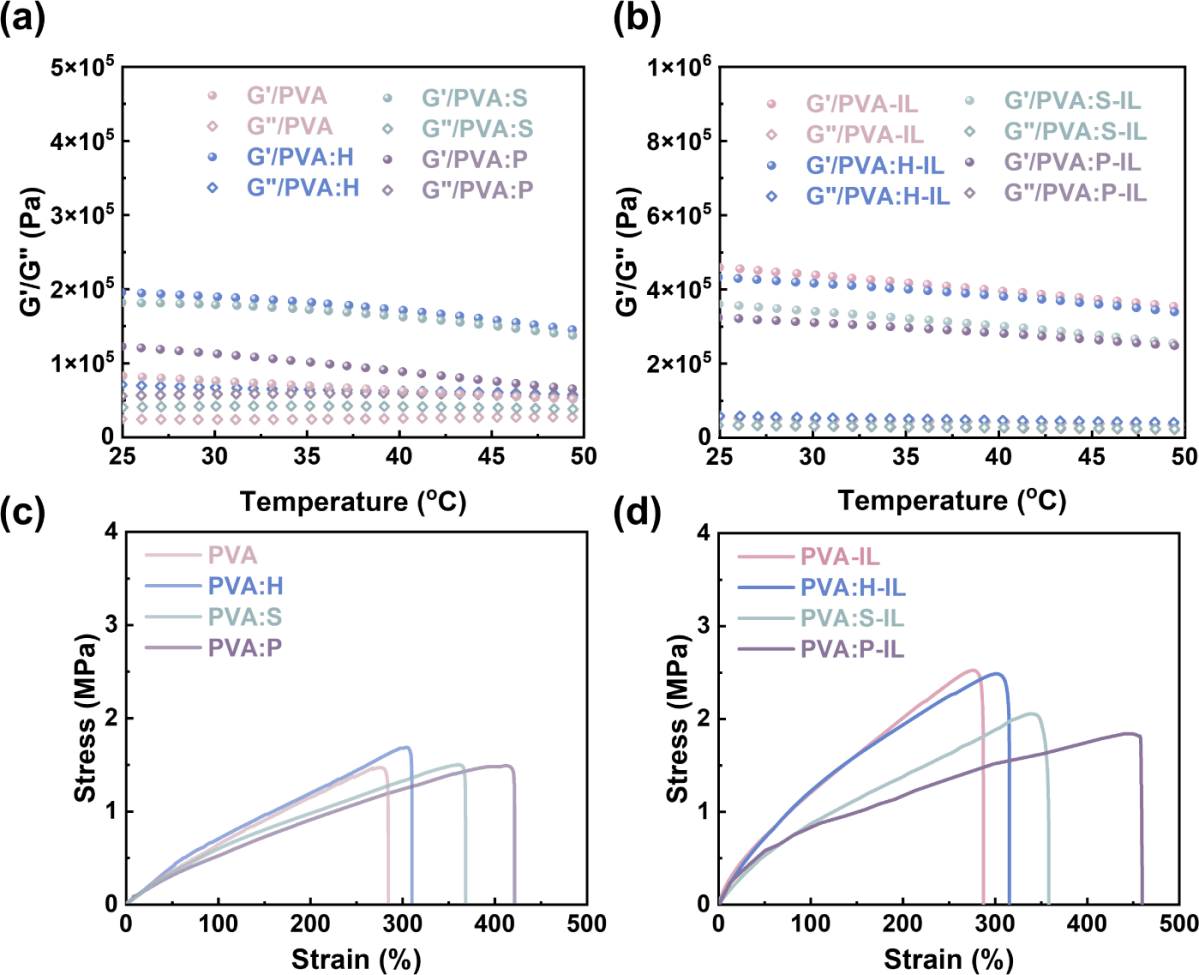
Mechanical and rheological properties of the PVA-based gels. Dynamic mechanical analysis showing the storage modulus (*G’*) and loss modulus (*G”*) of PVA-based (a) organogels and (b) ionogels. Tensile stress–strain curve of (c) organogels and (d) ionogels.

### Thermal Properties in PVA-Based Gels

To assess the thermal stability and behavior of the PVA-based organogels and ionogels, thermogravimetric analysis (TGA) and differential scanning calorimetry (DSC) were performed, with the results presented in Figure S6. As shown in Figure S6a,b, the PVA organogel exhibits greater weight loss and a lower decomposition temperature (*T*_d_) compared to the PVA ionogel. This phenomenon can be ascribed to the introduction of the IL that modulates molecular interactions within PVA, facilitating the formation of crystalline domains. Furthermore, as the Figure S6b, the introduction of the additives leads to a slight decrease in *T*_d_, which can be attributed to the reduction in crystallinity induced by the additives. These additives likely disrupt the ordered arrangement of PVA chains, weakening intermolecular interactions and thereby lowering the thermal stability of the material.^42^ Notably, among the tested additives, the P additive results in the greatest decrease in *T*_d_. This observation aligns well with the previous analysis manifesting that the P additive effectively modulates PVA crystallinity by interacting with hydroxyl groups and altering molecular packing.

The DSC results displayed in Figure S6c,d reveal that the incorporation of different additives significantly influences the thermal behavior of PVA-based organogels and ionogels. The PVA organogels show a much lower melting temperature *T*_m_ (85.45 °C) and crystallinity than those of dry neat PVA (*T*_m_ > 200 °C) because of solvation effect. The incorporation of the additives further decreases *T*_m_, especially for the P additive (*T*_m_ = 80.69 °C). After soaking in IL, *T*_m_ of the PVA-based ionogels significantly increases to ∼ 120 °C and the crystallinity also increases, indicating that IL can effectively promote crystallization of PVA in gels. The degree of crystallinity from DSC results (*Χ*_DSC_) is calculated according to equation S1- and summarized in Table S4. Among the additives, P exhibits the most pronounced decrease in *T*_m_ and crystallinity, from 126.08 °C and 5.61% of PVA-IL to 105.68 °C and 1.61% of PVA:P:IL. This suggests the strong ability of the P additive to disrupt hydrogen bonding and expand the amorphous regions of PVA. The DSC results align with the XRD and SAXS analyses that demonstrate a higher crystallinity of PVA-based ionogels than that of the PVA-based organogels and the same trend of reduction in crystallinity affected by the additives.

### Characterization of the i-TE Performance in PVA-Based Ionogels

Based on the analysis above, it can be inferred that ionogels exhibit distinct characteristics depending on the additives introduced. Subsequently, an investigation of the TE properties of the ionogel electrolyte with varying additives was conducted. The thermopower (*S*_i_) of the ionogels was evaluated in the vertical direction using a customized experimental setup. In this configuration, a controlled temperature gradient (Δ*T*) was applied to the hydrogels at two ends, which triggered the generation of a thermovoltage (Δ*V*). The *S*_*i*_ can subsequently be determined by finding the slope of the linear relationship between Δ*V* and Δ*T*. The experimental results, as depicted in Figure S7, underscore the pronounced *S*_*i*_ exhibited by the ionogels with different additives. Due to the high versatility of manufacturing ionogels, two different sizes (bulk and film) were fabricated to conduct vertical and horizontal i-TE tests. In the vertical measurement shown in Figure 7a, the *S*_i_ of the PVA-IL ionogels reaches 2.5 mV K^–1^, i.e., cation-induced (p-type) behavior. Additionally, the *S*_i_ increases to 5.6, 5.8, and 9.5 mV K^–1^ in PVA:H-IL, PVA:S-IL, and PVA:P-IL, respectively. This effect is similar to the results observed in the horizontal measurement in Figure 7b where the *S*_i_ values are 2.8 mV K^–1^ for PVA-IL ionogels, and 5.3, 5.5, and 9.2 mV K^–1^ for PVA:H-IL, PVA:S-IL, and PVA:P-IL, respectively. The linear fitting of Δ*V* with different Δ*T* values for ionogels is shown in Figure S7 and Table S5. All ionogels with two sizes show R-squared values above 0.99, i.e., the i-TE properties are relatively stable. More specifically, this stability is attributed to the high thermal stability, molecular compatibility, stable physical structure, and reduced volatility of the organic solvent and IL system.^22^ The illustration of *S*_i_ for PVA:X-IL shown in Figure 7c depicts the ion transport mechanism and suggests that introducing additives not only controls the crystallinity of the PVA polymer (resulting in an enlarged amorphous region) but also interacts with DCA anions (limiting transport to enhance the concentration gradient between the anions and cations). Furthermore, the EMIM cations possess chaotropic properties, which allow them to pass through amorphous regions more efficiently. The relationship between crystallization behavior can be characterized and confirmed through various analytical techniques such as SEM, XRD, and SAXS. These methods provide insights into the structural arrangement, crystalline phases, and microstructures of the gel-based materials. Furthermore, the interaction between the PVA polymer, additives, and IL can be demonstrated through DFT simulations as well as FT-IR and XPS.

**Figure 7:**
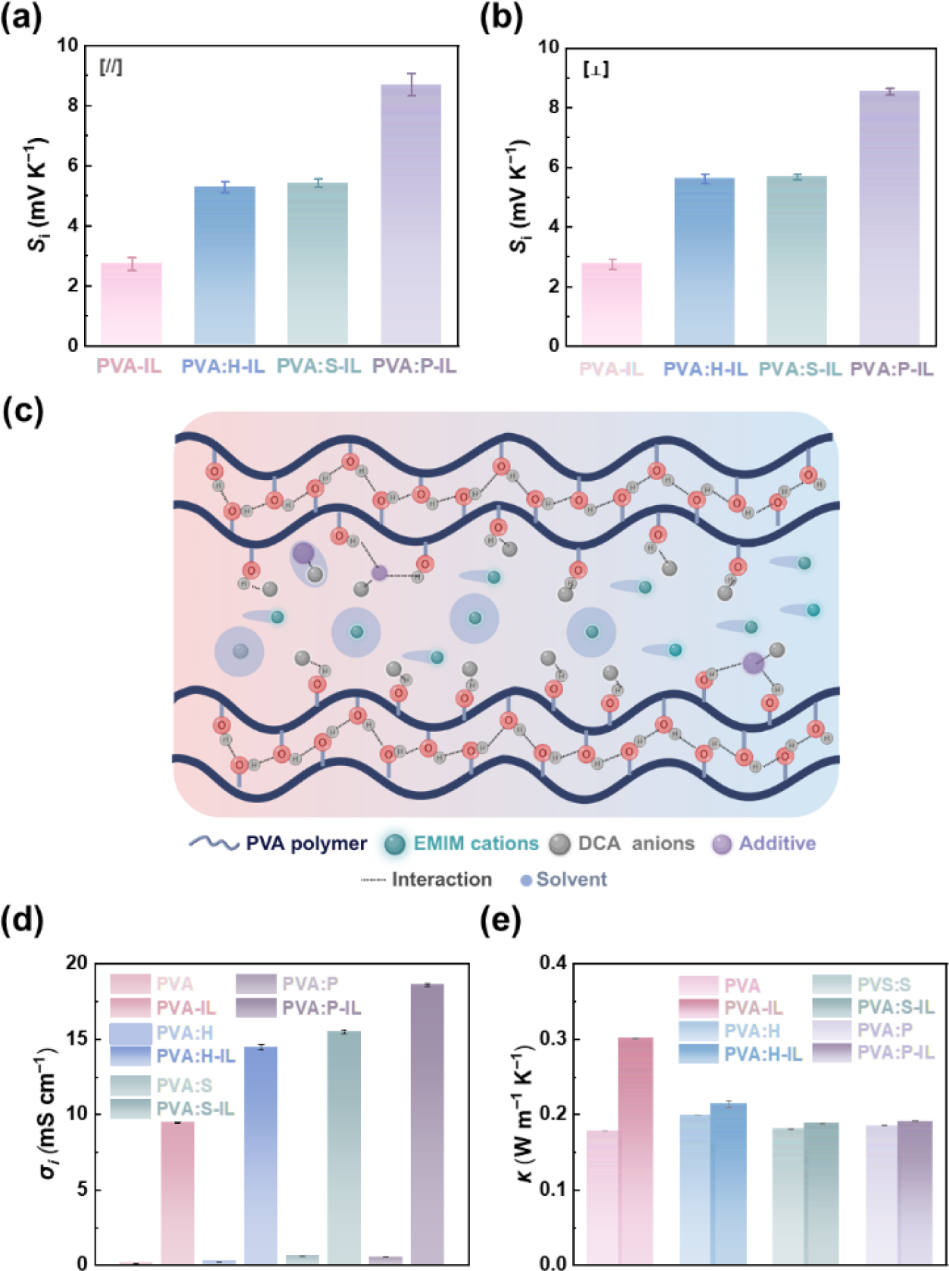
i-TE properties of PVA-based ionogels. (a) vertical and (b) horizontal thermopower measurements. (c) Illustration depicting ionic transport of the PVA:X-IL under a temperature gradient. (d) Ionic conductivity. (e) Thermal conductivity.

The ionic conductivity (σ_i_) of both organogels and ionogels was determined through AC impedance spectroscopy – see Figures 7c and S8. The organogels exhibit a σ_i_ below 1 mS cm^–1^ due to a lower concentration of the ion conductor. However, the σ_i_ substantially increases with ionic-liquid immersion. Notably, as shown in Table S6, when comparing the PVA-IL, the ionogels with additives show slight enhancements in the following order: PVA:H-IL, PVA:S-IL, and PVA:P-IL, respectively. The enhancement in σ_i_ is facilitated by the amorphous structure, which allows cations to move more freely.^43^ The PVA:P-IL with the highest amorphous regions exhibits the highest σ_i_, reaching 18.5 mS cm^–1^, which highlights its superior ion transport efficiency. The thermal conductivity (κ) shown in Table S6, which is another parameter relevant to the i-TE properties, is elucidated in Figure 7d. The organogels show lower κ values due to the discontinuation of lattice vibration between crystallinity and amorphous regions, which can lower κ.^44^ Among all organogels, PVA:P features more amorphous regions than other organogels, which results in a lower κ (0.18 W m^–1^ K^–1^). Upon IL immersion, all ionogels show increased κ values, e.g., from 0.18 W m^–1^ K^–1^ in PVA organogel to 0.3 W m^–1^ K^–1^ in PVA-IL, due to enhanced crystallinity. However, PVA:P-IL, which features larger amorphous regions, maintains a κ of 0.19 W m^–1^ K^–1^.

It is crucial to note that i-TE materials cannot be directly used in thermoelectric generators for heat harvesting. This limitation arises because ions cannot transport across the electrodes to the external circuit. Instead, these materials are more suited for applications in ITECs. The PVA:P-IL has excellent i-TE properties and emerges as a promising candidate for incorporation into ITECs. As demonstrated in a previous study,^9^ the Δ*V* during one thermal cycle can be divided into four distinct stages. In Figure 8a featuring the PVA:P-IL ionogel, during stage I, when a Δ*T* of 15 K is applied across two electrodes of an ITEC, EMIM cations accumulate at the cold side and generate a Δ*V* of – 136 mV after 13 min. In stage II, upon connecting an external load of 140 kΩ to the device, Δ*V* rapidly decreases due to charge screening via the external circuit. In stage III, when the Δ*T* is turned off, and the external load is disconnected, a positive Δ*V* arises. As the accumulated cations and anions retreat within the ionogels, the electrons and holes necessary for charge balance remain at the two electrodes. Note that this occurs as Δ*T* dissipates, which leads to a corresponding change in Δ*V* of approximately 130 mV. In stage IV, upon reconnecting the external load, the generated Δ*V* drops to nearly zero, indicating the discharge of the ITEC. Additionally, as shown in Figure 8b, the Δ*V* decay during stage II decreases with higher external resistance and causes a longer decay time. When the external load is 140 kΩ, the maximum power density of PVA:P-IL reaches 6.8 mW m^–2^ – see Figure 8c. These findings highlight the significant potential that PVA:P-IL has for the development of efficient ITECs.

**Figure 8:**
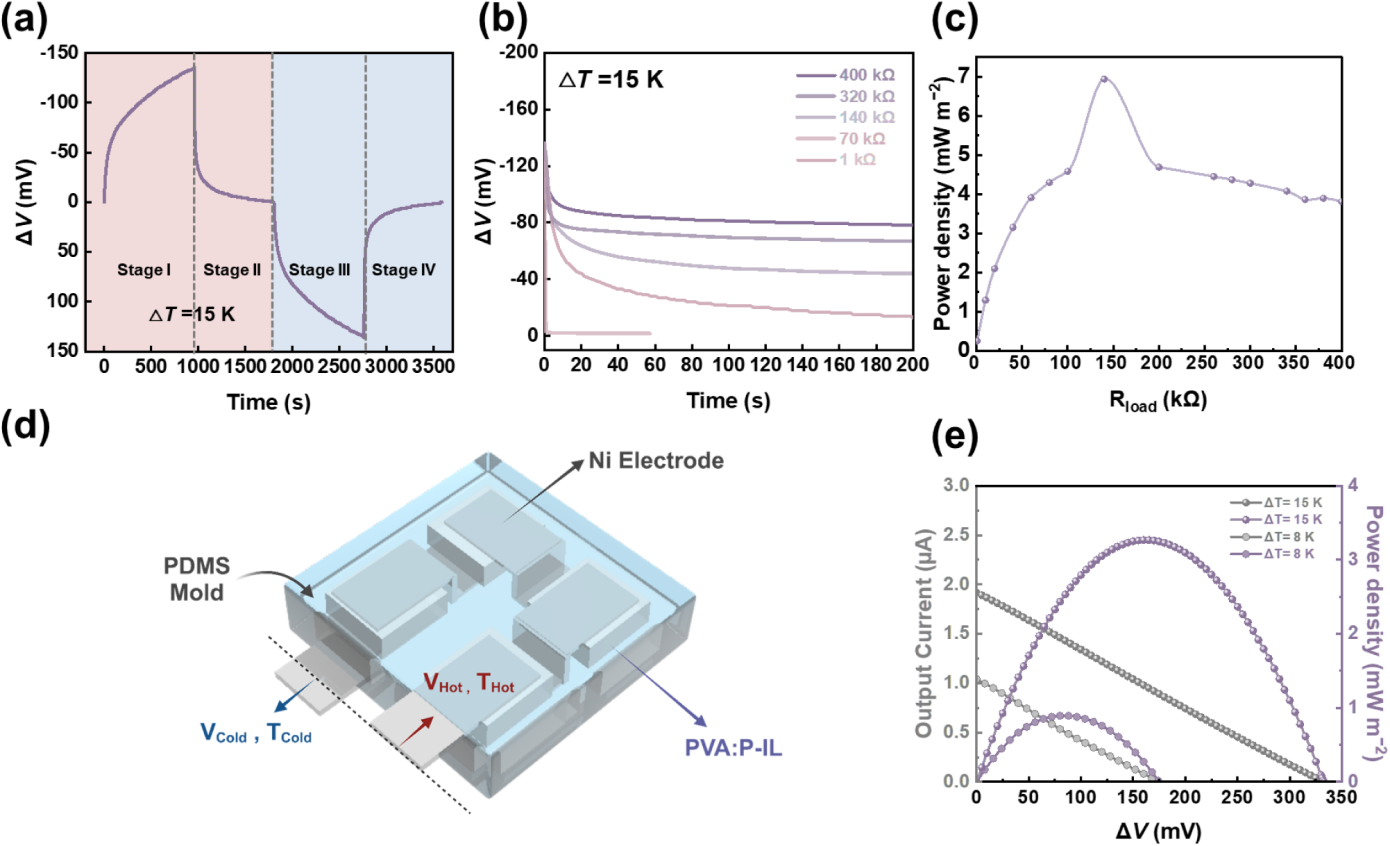
ITEC performance, including both unit cells and 4-leg modules. (a) Δ*V* and Δ*T* variations for the four-stage profile, (b) decay curves of Δ*V* under external loads with varying resistances (1, 70, 140, 320, and 400 kΩ) connected to the ITEC, (c) maximum power density supplied by the ITECs during stage II and stage IV for the different resistances associated with the external load. (d) Illustration of the 4-leg ITEC module, including the PDMS mold, Ni electrode, and PVA:P-IL ionogels, along with the hot and cold voltage and temperature and (e) their corresponding output current and power density for different Δ*T* values ranging from 8 and 15 K.

### Thermoelectric Module Analysis

Leveraging the high i-TE performance, excellent mechanical properties, and stability of the PVA:P-IL, an enhanced thermovoltage module array was developed. This array consists of four PVA:P-IL units that are interconnected by flexible Ni electrodes. This device configuration is schematically shown in Figure 8d and explained in detail in the Methods section. Each PVA:P-IL unit was sliced and placed in a PDMS mold with the dimensions 0.4 × 0.4 cm^2^ and a thickness of 1 cm. The module array was then encapsulated with thermoconductive tape on both the top and bottom surfaces as a package. The attached image also shows the Δ*V* of the PVA:P-IL i-TE module as a function of the loading current (*I*_load_) at different temperature differences (Δ*T* = 8 and 15 K). The voltage–current (*V*-*I*_load_) plots, represented by a black line, are linear for the PVA:P-IL devices, which confirms their stable resistance. The corresponding output power (*P*) can be calculated using the formula *p* = *V* × *I*_load_ and is shown in Figure 8e (purple line). At Δ*T* = 15 K, the module exhibits much higher power values compared to the Δ*T* = 8 K scenario. The maximum output power density (*ω*_*max*_) values are calculated using the formula: *ω*_*max*_ = *P*_*max/*_(*N* × *A*),^45^ where *P*_max_ is the maximum output power, *N* denotes the number of i-TE legs, and *A* represents the cross-sectional area of each leg. The *ω*_*max*_ values for Δ*T* of 8 and 15 K are 0.26 and 1.28 mW cm^–2^, respectively. These results suggest a significant potential for PVA:P-IL devices when it comes to the development of efficient thermoelectric modules to enhance Δ*V* and *ω*_*max*_.

This work introduces a novel additive-assisted approach to tailoring the crystallinity of PVA-based ionogels, optimizing the balance between mechanical strength, thermal stability, and thermoelectric performance. The incorporation of specific additives (H, S, P) effectively regulates polymer crystallization, improving ionic conductivity and thermopower. Consequently, our ionogel-based ITEC device achieves outstanding energy conversion efficiency, delivering a maximum power output of 6.8 mW m^–2^. These advancements establish our PVA-based ionogel as a strong candidate for next-generation flexible and wearable ionic thermoelectric applications.

## Conclusions

This study focused on the fabrication of organogels from commercially available PVA polymers, which are renowned for their high mechanical strength, environmental stability, and ability to withstand high temperatures in organic solvents (during operation). However, while introducing ionic liquids (ILs) enhances conductivity, it also significantly increases crystallinity, which adversely affects the i-TE properties. The primary objective of this research was to introduce various functionalized additives and conduct a comprehensive investigation into ion interactions and crystallization properties. By carefully controlling PVA crystallization, this work aimed to increase thermopower (*S*_i_), enhance ionic conductivity (σ_i_), and reduce thermal conductivity (κ). The resulting PVA:P-IL exhibits notable mechanical properties featuring a stress of 1.75 MPa and a strain of 460%. The introduction of various additives was found to improve the ionic thermoelectric (i-TE) properties substantially based on SEM and SAXS analyses that focused on the polymer microstructure. FT-IR and XPS were used to investigate the interactions within the PVA polymer, the IL [EMIM]­[DCA], and different additives deeply. Additionally, XRD and thermal analysis were employed to analyze the crystallinity of the PVA polymer.

The observed enhancement can be attributed to the effective control over the PVA crystallinity and an expansion of the amorphous regions, which allows the EMIM cation (with its chaotropic nature) to migrate through the amorphous regions of PVA toward the cold side. Furthermore, the P additive, with its electron-withdrawing groups, is expected to disrupt PVA aggregation and interact with DCA anions. This interaction increases the concentration gradient of both anions and cations between the hot and cold sides at different temperatures. In addition, the *S*_i_ of PVA:P-IL ionogels reached 9.5 mV K^–1^ while the PVA:P-IL ionogels demonstrated the highest σ_i_ values (reaching 17 mS cm^–1^). This improvement is due to the enlarged amorphous regions that increase ion exchange sites or channels, which improves ion transport efficiency. Compared to PVA-IL ionogels, the PVA:P-IL ionogels exhibited lower κ values (up to 0.19 W m^–1^ K^–1^). This reduction in κ was inferred to be due to the disruption of lattice vibrations between the crystalline and amorphous regions.

Moreover, an ionic thermoelectric capacitor (ITEC) device achieved a maximum power output of 6.8 mW m^–2^ for a load resistance of 140 KΩ, and a 4-pair thermoelectric module produced a voltage output of 0.33 V with a maximum power output of 2.4 mW m^–2^. The maximum output power density (*ω*_*max*_) values for Δ*T* of 15 K were 1.28 mW cm^–2^. These results demonstrate the high i-TE performance of the developed PVA-based ionogels with an additive system.

In conclusion, the strategic introduction of specific additives enhanced the i-TE properties of PVA:P-IL ionogels by controlling crystallinity and expanding the amorphous regions effectively. This synergistic effect increased *S*_i_, σ_i_, and it reduced κ. The exceptional performance of these materials in ITEC devices highlights their strong potential for applications, particularly for the efficient recovery of low-grade waste heat.

## Methods

### Materials

Poly­(vinyl alcohol) (PVA) (*M*_*w*_ = 146,000 ∼ 1,869,000, 99+% hydrolyzed), N,N–dimethylformamide (DMF) (≥99%), Dimethyl sulfoxide (DMSO) (anhydrous, ≥ 99.9%), and polydimethylsiloxane (PDMS, base: crosslinker = 10:1) were purchased from Sigma-Aldrich Co. (USA). 1-Ethyl-3-methylimidazolium dicyanamide ([EMIM]­[DCA]) and 2-carboxyphenylacetic acid (*M*_*w*_ = 180.16, > 99.0%) were obtained from TCI Co. (Japan). 2-Sulfobenzoic acid hydrate (*M*_*w*_ = 202.19, 98%) and 2-carboxyphenyl phosphate (*M*_*w*_ = 218.10, 98%) were purchased from Alfa Aesar Co. (USA). All chemicals were used as received and without further purification.

### Fabrication of the PVA and PVA:X Organogel and Iongel

A 10 wt % PVA solution was prepared by dissolving the polymer in a cosolvent mixture of DMF and DMSO at a volume ratio of 6:4. The dissolution was carried out at 110 °C under magnetic stirring for 1 h. For the fabrication of PVA organogels without additives, the prepared solution was directly cast into a Teflon or silicone molds at room temperature for 24 h allowed to polymerzation. For the fabrication of PVA organogels with additives, carboxyphenylacetic acid (H) or 2-sulfobenzoic acid hydrate (S) or 2-carboxyphenyl phosphate (P), the additives were added to the PVA solution in a weight ratio of PVA to additive = 3:1. The mixture was then stirred for an additional 1 h to ensure uniform distribution of the additives. Finally, the solution was cast into a Teflon or silicone mold at room temperature for 24 h allowed to polymerzation. All the gels retain 90% of residual solvents.

The ionogel was obtained by soaking the organogel into ionic liquid ([EMIM]­[DCA]) for 2 days. Initially, the prefabricated organogel was soaked in the IL for 1 day to start the absorption process. Then, the IL was replaced, and the organogel was soaked for an additional day to ensure complete saturation with the IL.

### Thermoelectric Measurements

The thermopower (*S*_*i*_) of the ionogels was assessed within a custom-designed experimental setup maintained at 40% relative humidity. For the vertical (or horizontal) measurement, two platinum (Pt) electrodes, each measuring 1.5 × 1.5 cm^2^, were securely affixed to the upper and lower (or left and right) surfaces of the two end stages of the 0.5 × 0.5 × 0.3 cm^3^ (or 1 × 2 × 0.02 cm^3^) ionogels. The temperature gradient (Δ*T*) across the ionogels was controlled as follows: the temperature in the hot section was regulated within the range of 25 to 35 °C using a customized temperature controller, while the temperature in the cold section was held constant at 25 °C through the use of a water bath. Temperature measurements were carried out by placing two thermocouples between the hot side and the cold side. The voltage differences under each Δ*T* were recorded using a Keithley 2400A sourcemeter. To determine the *S*_*i*_, a linear regression analysis was performed on 6 to 8 data points. Furthermore, the *S*_*i*_ value for each ionogel was calculated based on data collected from five identically fabricated devices. The ionic conductivity (*σ*_*i*_) of the gels was determined by placing the samples between two Pt electrodes and assessing the ionic resistance through electrochemical impedance spectroscopy (EIS), conducted using a BioLogic SP-50e instrument with a voltage amplitude of 5 mV and a frequency range from 1 MHz to 10 mHz. Then *σ*_*i*_ was calculated based on the formula *σ*_*i*_ = *d*/(*R* × *A*), where *d* represents the thickness of the gels (0.3 cm), *A* is the cross-sectional area (0.5 × 0.5 cm^2^), and *R* is the bulk resistance. Thermal conductivity (κ) measurement was conducted using a Hot Disc TPS 2500S thermal constant analyzer with the standard module, employing the transient plane source (TPS) method.

### Thermoelectric Module Fabrication and Meausurement

The thermoelectric device was fabricated using a PVA:P-IL ionogels integrated into a (polydimethylsiloxane) PDMS mold. The PDMS mold was created from a 10:1 weight ratio mixture of base elastomer and curing agent, designed in a square shape with overall dimensions of 3 × 3 cm^2^, and featured four 1 cm thick holes, each meaursing 0.4 × 0.4 cm^2^. The mold was cured at 80 °C for 24 h to achieve the desired shape. Four pieces of PVA:P-IL, each measuring 0.4 × 0.4 × 1 cm^3^, were precisely cut and positioned within the holes. To create the connected electrodes, a zigzag pattern was formed by folding a Ni film. Thermal tape was strategically placed on the top and bottom surfaces of the module, while the both sides were covered with polyimide (PI) tape, effectively enclosing the entire module structure.

## Supplementary Material



## Notes

Further details of the preparation process and various compounds for gels, FT-IR spectra, TGA patterns, DSC patterns, dynamic mechanical measurement, the SAXS spectra­(PDF)
