# Supplementary material for: Enhanced Thermoelectric Performance of PVA-Based Ionogels: Tailoring Crystallinity via Additives for Advanced Waste Heat Recovery
Source: ACS Appl Mater Interfaces. 2025 Jun 21;17(26):38545–57. doi: 10.1021/acsami.5c08724 (PMC12232262; doi:10.1021/acsami.5c08724)
Supplement: Supplementary file 1 [file am5c08724_si_001.pdf]

Supporting Information

Enhanced Thermoelectric Performance of PVA-Based Ionogels: Tailoring Crystallinity via Additives for Advanced Waste Heat Recovery

Ling-Chieh Lee,[a] Shao-Huan Hong,[a] Min-Su Kim,[b] U-Ser Jeng,[c] Chia-Hsin Wang,[c] Shih-Huang Tung,[d] Keun Hyung Lee,[b] Cheng-Liang Liu[a,e]*

[a] Department of Materials Science and Engineering, National Taiwan University, Taipei 10617, Taiwan

[b] Department of Chemistry and Chemical Engineering, Inha University, Incheon 22212, Republic of Korea

[c] National Synchrotron Radiation Research Center, Hsinchu 30076, Taiwan

[d] Institute of Polymer Science and Engineering, National Taiwan University, Taipei 10617, Taiwan

[e] Advanced Research Center for Green Materials Science and Technology, National Taiwan University, Taipei 10617, Taiwan

*Corresponding author. Email: liucl@ntu.edu.tw (C.-L.L.)

KEYWORDS. ionic thermoelectrics, thermopower, ionic liquid, ionogel, low-grade heat harvesting

Characterization. Fourier transform infrared spectroscopy (FT-IR, TWO FT-IR L16000F, PerkinElmer Spectrum) was conducted on the organogel or ionogel to investigate the interactions between ions and functional groups of polymers. The crystallization behavior of PVA was analysed using X-ray diffraction (XRD) measurement conducted on a Rigaku MiniFlex instrument, covering 2θ range of 5~60°. The thermogravimetric analysis (TGA) of the gels was conducted using TA Instrument TGA-55 under nitrogen flow and with the heating rate of 10 °C min^{-1}. Differential scanning calorimetry (DSC) of gels was carried out under nitrogen on TA Instrument Discovery DSC-25 with the scanning rate of 10 °C min^{-1}). The crystallinity of PVA-based organogels and ionogels from DSC patterns could be calculated as

$$X_{\mathrm{DSC}} = \frac{H_{\mathrm{crystalline}}}{H^{\circ}_{\mathrm{crystalline}}} \times 100\%$$

(S1)

where $H_{\mathrm{crystalline}}$ represents the enthalpy for melting the crystalline per unit mass of PVA-based oroganogels and ionogels. The $H_{\mathrm{crystalline}}$ was calculated by the integration of the melting point of PVA-based gels on an endothermic curve. The $H^{\circ}_{\mathrm{crystalline}}$ = 138.6 J g^{-1} is the enthalpy of 100 wt.% crystalline PVA at the equilibrium melting point.[1,2] Mechanical analyses of the gels, with dimensions of 1 cm in width, 2 cm in length, and 0.2 cm in thickness, were conducted using a universal testing machine (EZ-Test, SHIMADZU) at a constant speed of 5 mm mim^{-1}. The rheological behaviour of the hydrogels was assessed using a rheometer (HR-2 system, TA Instrument) with a temperature range of 20 to 50 °C, varying at a rate of 5 °C min^{-1}. The ESP calculations were

performed using the Gaussian 09 program package, and the binding energies were calculated using DFT at the B3LYP/6-311G + (d, p) level. The morphology of the gels was visualized using scanning electron microscopy (SEM, S-4800, HITACHI). The gels underwent a freeze-forming process followed by freeze-drying to preserve their initial structure, and then a layer of gold was applied to enable surface examination. To determine the elemental composition, energy-dispersive X-ray spectroscopy (EDS) was performed using a QUANTAX Annular XFlash® QUAD FQ5060 instrument. Small-angle X-ray scattering (SAXS) and X-ray photoelectron spectroscopy (XPS) measurements were conducted at the National Synchrotron Radiation Research Center (NSRRC) in Taiwan, using Beamlines TPS 13A and TLS 24A1, respectively. During the XPS analysis, the chamber was maintained at a pressure of approximately 0.05 mbar to ensure the presence of water vapor during the measurement.

SAXS measurement The Beaucage unified equation was employed, offering a versatile framework for modeling scattering from a wide range of polymer structures, including polymer coils, polydisperse spheres, and various types of aggregates. This approach enables a comprehensive characterization of the multi-level structure of the gels and the various degrees of aggregation present within the material.

$$I(q) \cong I_{bkg} + \sum_{i=1}^{N} \left\{ G_i exp\left(-\frac{q^2 R_{gi}^2}{3} \right) + B_i exp\left(-\frac{q^2 R_{g(i+1)}^2}{3} \right) \times \left\{ \frac{\left[erf\left(\frac{q R_{gi}}{\sqrt{6}} \right) \right]^3}{q} \right\}^{P_i} \right\} \tag{S2}$$

In this equation (S2), G_i represents Guinier's pre-exponential factor, while B_i corresponds to the power law pre-exponential factor. R_{gi} stands for the radius of gyration, and P_i denotes the fractal dimension. In this context, i is associated with different levels of structural organization: i = 1 corresponds to the global structure and i = 2 signifies the characterization of local structures.

Table S1. Ionic Thermoelectric properties (i-TE) of PVA:P-IL ionogels compared to other ionogels and i-TE materials.

Gel	Solvent	IL	S_i (mV K^{-1})	σ_i (mS cm^{-1})	Stress (MPa)	Strain (%)	Ref.
PVDF-HFP	Acetone+ 5% ethonal	EMIM:DCA	25.4	17.6	-	-	3
PVDF-HFP	Acetone	EMIM:DCA	26.1	6.7	-	-	4
PVDF-HFP /PEG	Water	Emim:TFSI	14	0.8	-	-	5
PVDF-HFP	Acetone	EMIM:OTf	6.5	13.2	0.002	493	6
WPU	Water	EMIM:DCA	34.5	8.4	0.63	156	7
PU	Water	EMIM:DCA	25.6	12.8	1.61	300	8
PVA	Water	EMIM:DCA	4.67 (50 wt.% IL)	12.5 (50 wt.% IL)	0.64 (40 wt.% IL)	526 (40 wt.% IL)	9
**PVA**	**DMF:DMSO**	**EMIM:DCA**	**8**	**17.5**	**1.7**	**460**	This work

Table S2. Degree of crystallinity of organogels and ionogels. The degree of crystallinity is calculated from the deconvoluted XRD pattern.

Sample	Degree of Crystallinity (%)
PVA	12.44
PVA-H	11.91
PVA-S	11.29
PVA-P	10.15
PVA-IL	20.33
PVA:H-IL	19.82
PVA:S-IL	15.23
PVA:P-IL	12.39

Table S3. The fitting parameters of the two-level (organogels) ang three-level (ionogels) Beaucage model applied to the SAXS data obtained from the organogels and ionogels.

Sample	R_{g1} (nm)	P_1	R_{g2} (nm)	P_2
PVA	44.6	~2.0	4.5	~4.0
PVA:H	32.1		4.0	
PVA:S	34.8		3.8	
PVA:P	33.7		3.8	
PVA-IL	123.1		4.9	
PVA:H-IL	58.1		4.9	
PVA:S-IL	46.8		4.5	
PVA:P-IL	45.4		4.1	

Table S4. The melting point and degree of crystallinity of organogels and ionogels. The melting point and degree of crystallinity are calculated from the DSC pattern.

Sample	T_m (°C)	Degree of Crystallinity (%)
PVA	85.45	0.11
PVA-H	85.30	0.05
PVA-S	84.48	0.03
PVA-P	80.69	0.02
PVA-IL	126.08	5.61
PVA:H-IL	124.39	4.56
PVA:S-IL	111.64	3.35
PVA:P-IL	105.68	1.61

Table S5. The thermopower (S_i) of ionogels in vertical and horizontal measurements.

Vertical	S_i (mV K^{-1})
PVA-IL	2.5
PVA:H-IL	5.6
PVA:S-IL	5.8
PVA:P-IL	9.5
**Horizontal**	**S_i (mV K^{-1})**
PVA-IL	2.8
PVA:H-IL	5.3
PVA:S-IL	5.5
PVA:P-IL	9.2

Table S6. The ionic conductivity (σ_i) and thermal conductivity (κ) values of organogels and ionogels.

Sample	σ_i (mS cm^{-1})	κ (W m^{-1} K^{-1})
PVA	0.10	0.18
PVA:H	0.22	0.20
PVA:S	0.65	0.18
PVA:P	0.50	0.19
PVA-IL	9.45	0.30
PVA:H-IL	14.36	0.21
PVA:S-IL	15.44	0.18
PVA:P-IL	18.50	0.19

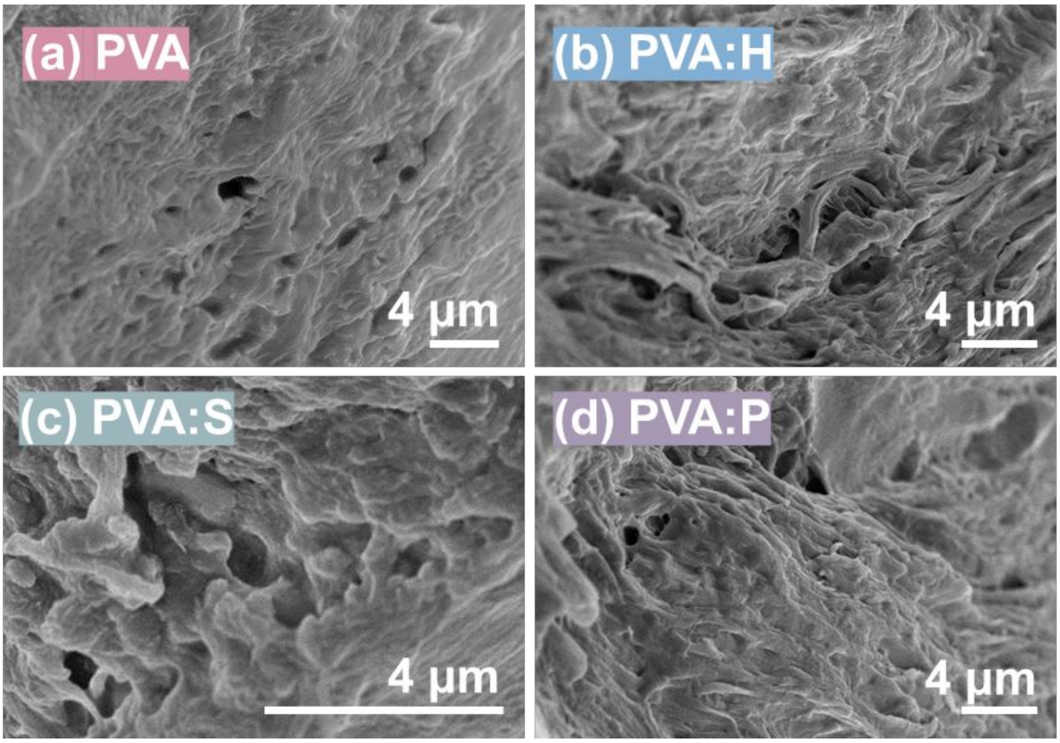

Figure S1. The cross-sectional morphologies of the organogels (a) PVA, (b) PVA:H, (c) PVA:S, and (d) PVA:P.

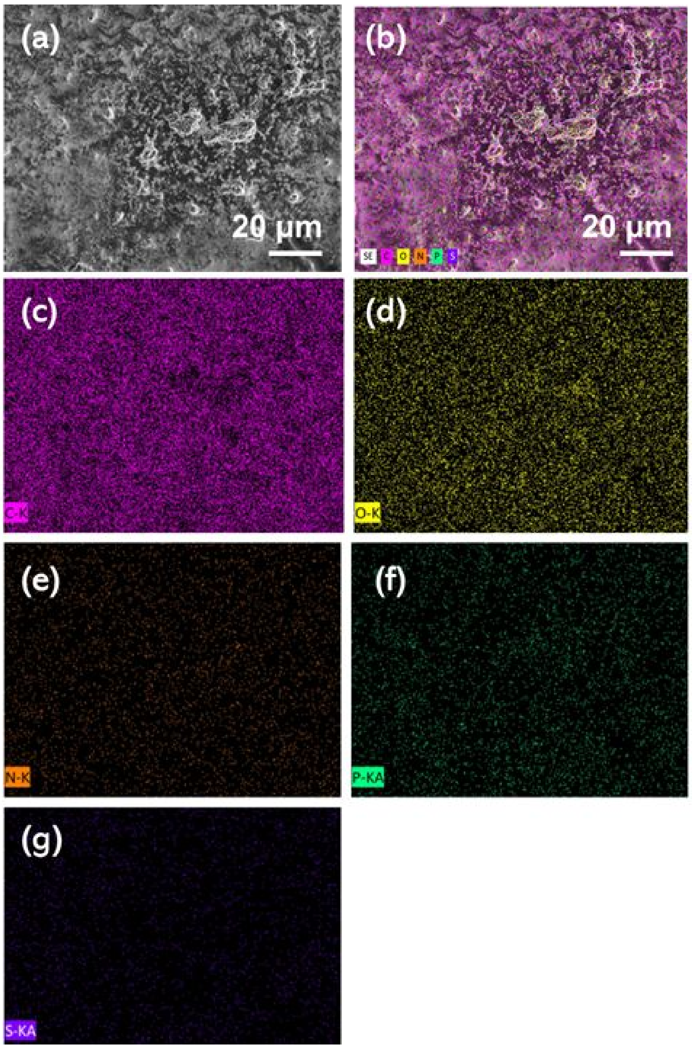

Figure S2. Lower magnification SEM micrograph and EDS elemental mapping of PVA:P-IL. (a) SEM image, (b) Combined EDS map, (c) Carbon (C, dark purple), (d) Oxygen (O, yellow), (e) Nitrogen (N, orange), (f) Phosphorus (P, green), (g) Sulfur (S, white purple).

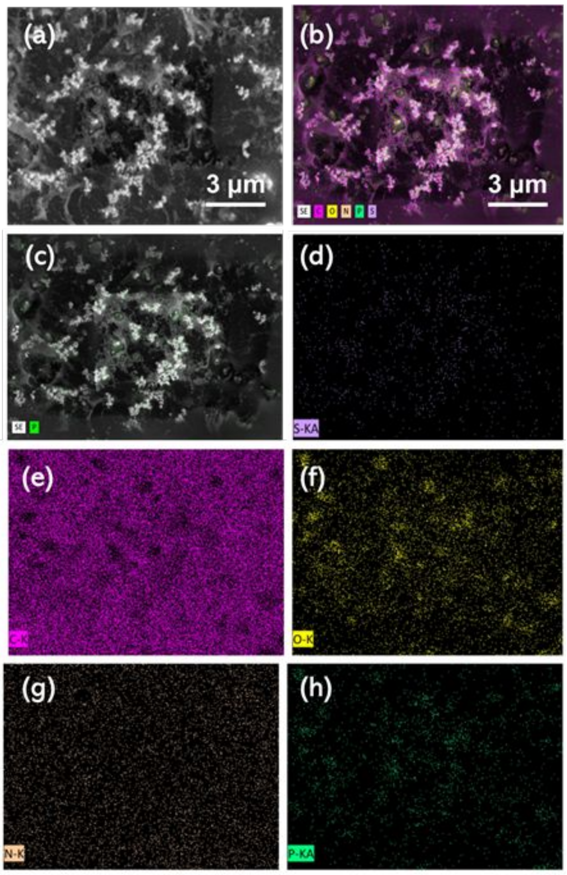

Figure S3. Higher magnification SEM micrograph and EDS elemental mapping of PVA:P-IL. (a) SEM image, (b) Combined EDS map, (c) Highlighted region in SEM image with phosphrous, (d) Sulfur (S, white purple), (e) Carbon (C, dark purple), (f) Oxygen (O, yellow), (g) Nitrogen (N, orange), (h) Phosphorus (P, green).

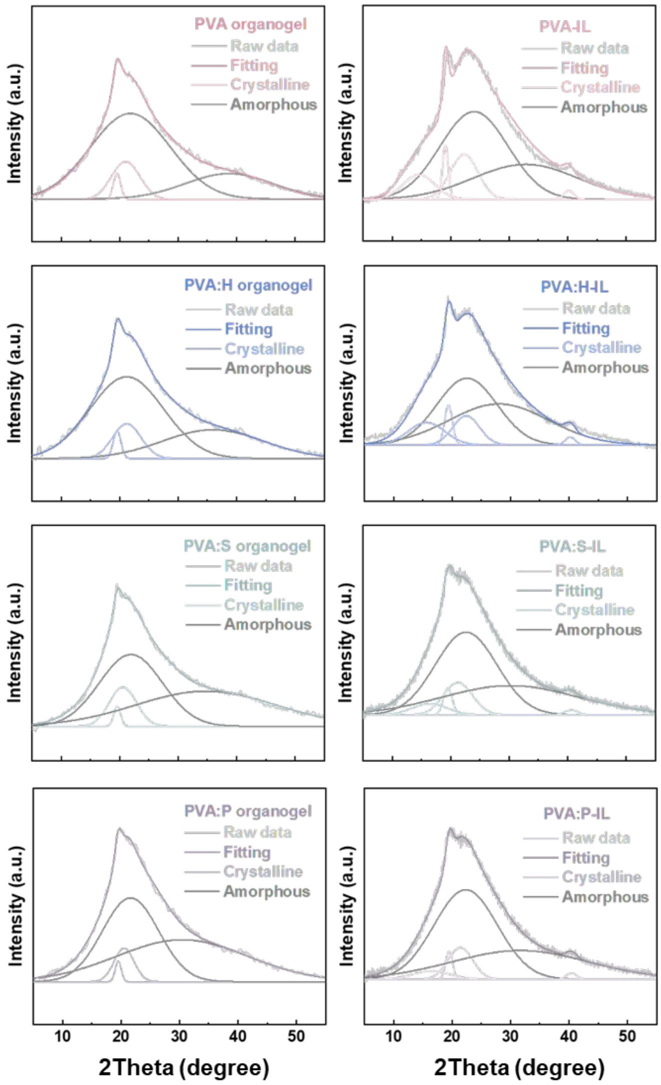

Figure S4. Fitting XRD deconvolution for organogels (right column) and ionogels (left column).

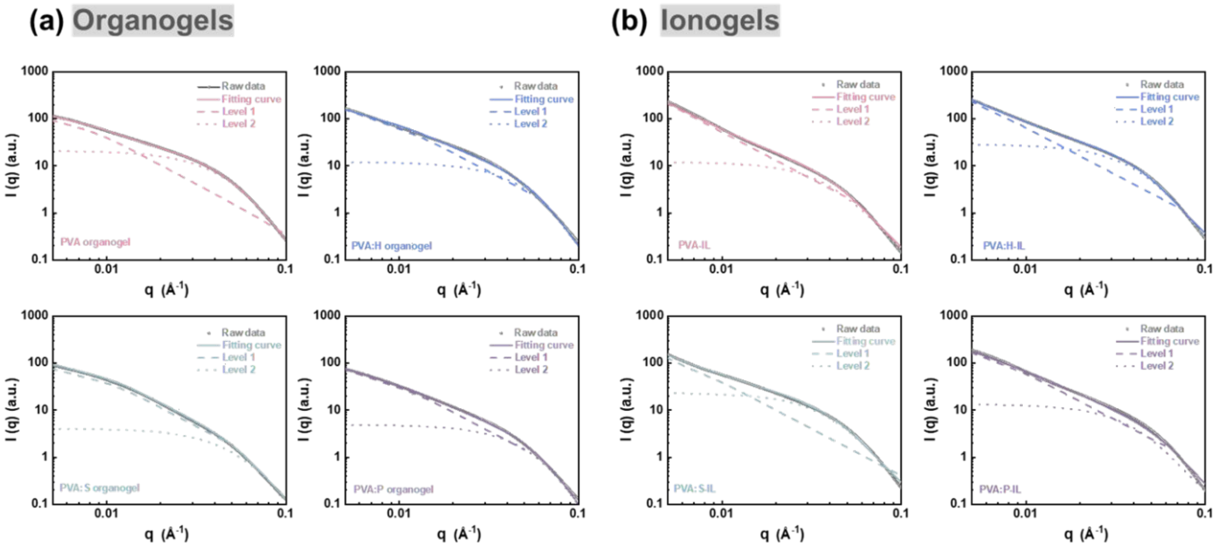

Figure S5. SAXS patterns of the (a) organogels and (b)ionogels. The dashed line represents the respective 1st and 2nd fitting results based on the Beacuage model.

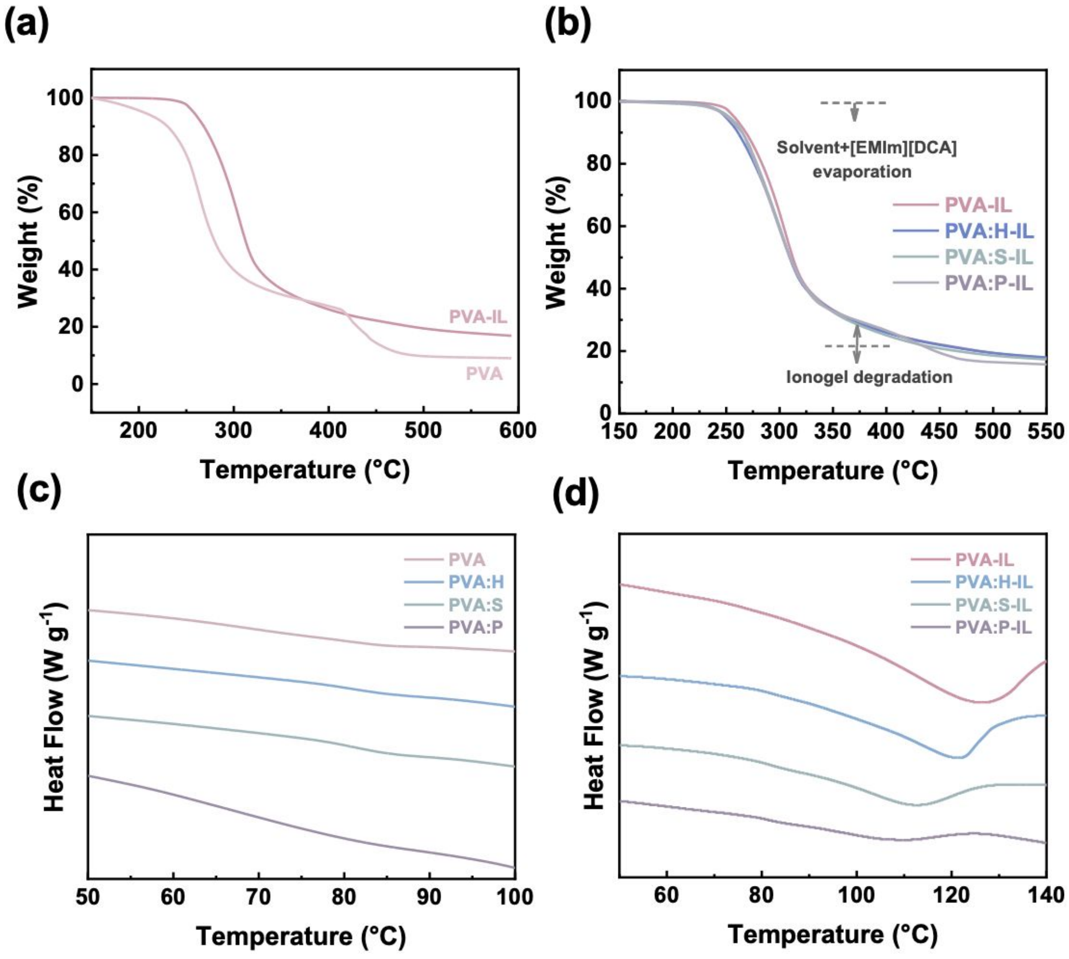

Figure S6. (a) TGA thermogram of PVA organogel and PVA-IL ionogel. (b) TGA thermogram of PVA-IL, PVA:H-IL, PVA:S-IL, PVA:P-IL ionogels. (c) DSC thermograms for the PVA, PVA:H, PVA:S, PVA:P organogels. (d) DSC thermograms for the PVA-IL, PVA:H-IL, PVA:S-IL, PVA:P-IL ionogels.

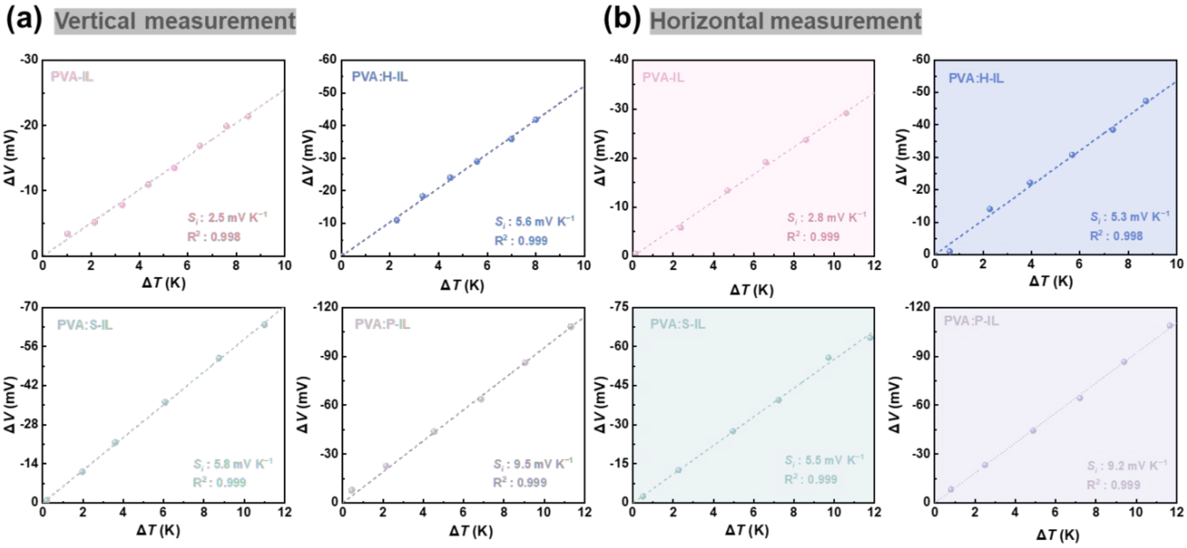

Figure S7. Linear fitting of ΔV against various ΔT values for (a) vertical measurement and (b) horizontal measurement.

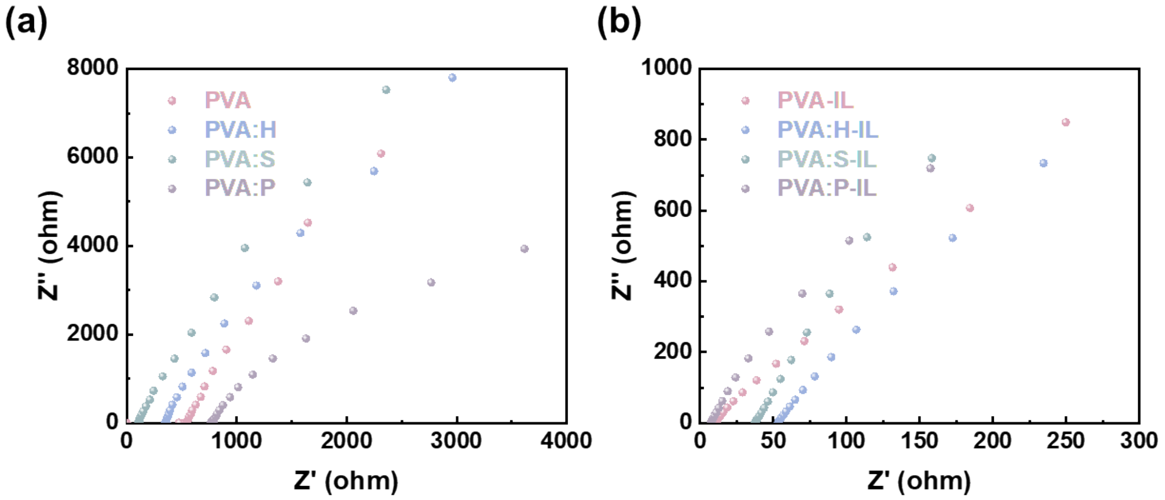

Figure S8. Ionic conductivity (σ_i) of observed in (a) organogels and (b) ionogels.

Reference

(1) Lin, S.; Liu, X.; Liu, J.; Yuk, H.; Loh, H.-C.; Parada, G. A.; Settens, C.; Song, J.; Masic, A.; McKinley, G. H.; Zhao, X. Anti-Fatigue-Fracture Hydrogels. *Sci. Adv.* **2019**, *5*, eaau8528.

(2) Peppas, N. A.; Merrill, E. W. Differential Scanning Calorimetry of Crystallized PVA Hydrogels. *J. Appl. Polym. Sci.* **1976**, *20*, 1457-1465.

(3) Cheng, H.; He, X.; Fan, Z.; Ouyang, J. Flexible Quasi-Solid State Ionogels with Remarkable Seebeck Coefficient and High Thermoelectric Properties. *Adv. Energy Mater.* **2019**, *9*, 1901085.

(4) Liu, Z.; Cheng, H.; He, H.; Li, J.; Ouyang, J. Significant Enhancement in the Thermoelectric Properties of Ionogels through Solid Network Engineering. *Adv. Funct. Mater.* **2022**, *32*, 2109772.

(5) Zhao, D.; Martinelli, A.; Willfahrt, A.; Fischer, T.; Bernin, D.; Khan, Z. U.; Shahi, M.; Brill, J.; Jonsson, M. P.; Fabiano, S.; Crispin, X. Polymer Gels with Tunable Ionic Seebeck Coefficient for Ultra-Sensitive Printed Thermopiles. *Nat. Commun.* **2019**, *10*, 1093.

(6) Akbar, Z. A.; Malik, Y. T.; Kim, D.-H.; Cho, S.; Jang, S.-Y.; Jeon, J.-W. Self-Healable and Stretchable Ionic-Liquid-Based Thermoelectric Composites with High Ionic Seebeck Coefficient. *Small* **2022**, *18*, 2106937.

(7) Fang, Y.; Cheng, H.; He, H.; Wang, S.; Li, J.; Yue, S.; Zhang, L.; Du, Z.; Ouyang, J. Stretchable and Transparent Ionogels with High Thermoelectric Properties. *Adv. Funct. Mater.* **2020**, *30*, 2004699.

(8) Xu, J.; Wang, H.; Du, X.; Cheng, X.; Du, Z.; Wang, H. Highly Stretchable PU Ionogels with Self-Healing Capability for a Flexible Thermoelectric Generator. *ACS Appl. Mater. Interfaces* **2021**, *13*, 20427-20434.

(9) Huang, Y.; Zhao, X.; Ke, J.-L.; Zha, X.-J.; Yang, J.; Yang, W. Engineering Nanoscale Solid Networks of Ionogel for Enhanced Thermoelectric Power Output and Excellent Mechanical Properties. *J. Chem. Eng.* **2023**, *456*, 141156.

`
